# Oxygen doped graphitic carbon nitride nanosheets for the degradation of organic pollutants by activating hydrogen peroxide in the presence of bicarbonate in the dark†

**DOI:** 10.1039/d0ra07893j

**Published:** 2020-12-24

**Authors:** Tian-Jiao Jiang, Chao Xie, Huai-De Peng, Bo Lei, Qing-Qing Chen, Gang Li, Cai-Wu Luo

**Affiliations:** School of Resource Environmental and Safety Engineering, University of South China 421000 China luocaiwu00@126.com +86-734-8282345; State Key Laboratory of Safety and Health for Metal Mines, Sinosteel Maanshan General Institute of Mining Research Co., Ltd 243000 China; Key Laboratory of Clean Energy Material, LongYan University 364012 China; Research Center for Eco-Environmental Sciences, Chinese Academy of Sciences 100085 China

## Abstract

The development of novel wastewater treatment processes that use heterogeneous catalysts to activate hydrogen peroxide (H_2_O_2_) with bicarbonate (HCO_3_^−^) has been a subject of great interest in recent years; however, significant challenges remain, despite research into numerous metal-based catalysts. The work presented herein employed oxygen-doped graphitic carbon nitride (O/g-C_3_N_4_) as a non-metal catalyst for activating H_2_O_2_ in the presence of HCO_3_^−^, and this method represented the first system capable of removing organic pollutants in the dark, to our knowledge. The catalysts were characterized using several microscopic imaging, spectroscopic, electrochemical, and crystallographic techniques, as well as N_2_-physorption procedures. Analysis of the results revealed that the O/g-C_3_N_4_ catalyst possessed a high specific surface area and many defect sites. Various operational parameters, including the relative amounts of HCO_3_^−^, H_2_O_2_, and O/g-C_3_N_4_, were systemically investigated. A clear performance enhancement was observed in the degradation of organic contaminants when subjected to the HCO_3_^−^–H_2_O_2_–O/g-C_3_N_4_ system, and this result was ascribed to the synchronous adsorption and chemical oxidation processes. The novel system presented herein represented a new water treatment technology that was effective for removing organic contaminants.

## Abstract 2

Bicarbonate enhanced the degradation of organic pollutants over oxygen doped graphitic carbon nitride nanosheets in the presence of hydrogen peroxide. It occurred the both on the surface of the catalyst and the reaction solution.

## Graphical abstract

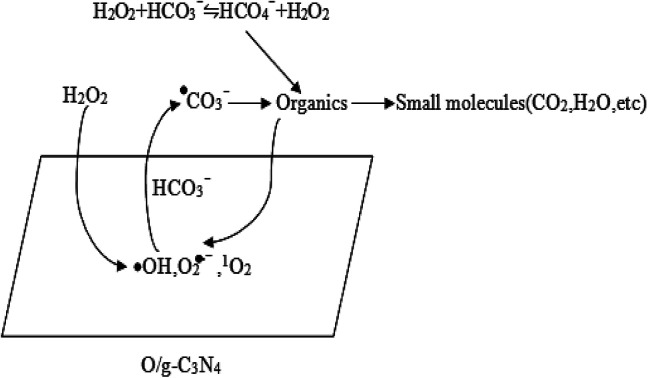

## 1. Introduction

The bicarbonate anion, HCO_3_^−^, is relatively nontoxic and is common in natural water ecosystems.^1^ For example, its concentration is known to reach 50–200 ppm in biological systems, and it may be presented at 14.7–25 mM in humans. It is worthwhile to note that HCO_3_^−^ is always generated from CO_2_ as a main product of advanced oxidation processes, which are typically impractical for certain applications. Directly introducing HCO_3_^−^ into a solution containing H_2_O_2_ often inhibits the degradation reaction; however, a few cases demonstrate that this process can promote the removal of organic pollutants.^2–11^ Despite this promising result, the huge required dosage is one of the main challenges associated with this approach because it significantly increases the cost of the treatment. To reduce this input cost, various transition metal ions, such as cobalt(ii)^12–18^ and copper(ii),^19,20^ have been tested as homogeneous catalysts. The results of those studies demonstrate that such metal catalysts accelerate the degradation of organic pollutants and distinctly reduce the required concentrations of HCO_3_^−^ and H_2_O_2_. However, it is extremely difficult to recover the catalyst from the product solution, which is a significant drawback of this approach. As an alternative, various heterogeneous catalysts have been carefully developed to avoid such problems. To date, these systems have involved metal-based materials, such as Co-based^21–25^ and Cu-based catalysts.^26–28^ For example, Guo *et al.*^23^ reported an S-modified CoFe_2_O_4_ catalyst, which degraded acid orange II in a reactive system containing HCO_3_^−^ and H_2_O_2_. Similarly, Pi *et al.*^25^ showed that their Co_*x*_Mn_3−*x*_O_4_ material was an effective catalyst for removing chlorophenols in the presence of a naturally-occurring concentration of HCO_3_^−^. Despite these advances, the leaching of toxic metal ions must be confronted during these reactions, because this phenomenon easily caused a second contamination and may reduce the stability of the catalyst. Therefore, other useful strategies have been explored.^29,30^ For example, Pétrier *et al.*^29^ adopted sonochemical technology to enhance the degradation of bisphenol in the presence of HCO_3_^−^. However, the required complex equipment input increased the cost. Therefore, in order to replace metal-based heterogeneous methods, there is a pressing need to develop simple non-metal-based heterogeneous catalysts that are inexpensive and green, and have excellent activity in combination with H_2_O_2_ and HCO_3_^−^.

Among non-metal heterogeneous catalysts, graphitic carbon nitride is an advantageous choice because it is safety, low cost, and has high stability. Importantly, it exhibits excellent catalytic performance under visible light illumination. Therefore, this type of catalyst has been applied for various purposes, including hydrogen production,^31–34^ but there are limited reports describing its activity for dark Fenton-like reaction. Cui *et al.*^32^ reported the degradation of organic dyes using g-C_3_N_4_ in the presence of H_2_O_2_; however, this system showed hardly any catalytic activity in the dark. To attain the aforementioned target activity, it is necessary to first explore the catalytic sites in g-C_3_N_4_ to evaluate H_2_O_2_ decomposition into reactive oxygen species (ROS). In general, this type of catalyst is characterized by extremely low specific surface area, which plays a vital role in the adsorption of organic pollutants. This factor significantly hinders the broad application of such catalysts in industry. To overcome this challenge, our group^34^ developed a novel g-C_3_N_4_ catalyst using oxygen modification. This O/g-C_3_N_4_ material possessed a huge specific surface area and exhibited some catalytic activity for the dark Fenton-like reaction. To further improve its catalytic performance, further system modification is necessary. As mentioned above, it can be expected that introducing HCO_3_^−^ to the system should strengthen its capability to degrade organic pollutants in wastewater. This modification creates a weakly alkaline environment, which is favorable for decomposing H_2_O_2_ into ROS.^21^ However, it may generate new radicals, such as CO_3_˙^−^. The surface of g-C_3_N_4_ has a negative charge under near-alkaline conditions; therefore, it generates a counteracting force whereby HCO_3_^−^ leaves the surface of the catalyst, thus inevitably capturing ROS, such as ˙OH radicals. Once the ˙OH radicals enter the solution, they are immediately captured by HCO_3_^−^ to produce CO_3_˙^−^ radicals, which have a longer lifetime. A literature survey^35,36^ confirmed that CO_3_˙^−^ radicals could directly remove organic pollutants. Still, the relationships between the specific surface area, nitrogen defective sites, and catalytic activity must be further elucidated, especially in terms of the dark Fenton-like reaction.

Herein, we describe a novel treatment technique for removing organic pollutants during dark Fenton-like reaction. To our knowledge, this is the first time that O/g-C_3_N_4_ has been used as a heterogeneous catalyst in a system containing HCO_3_^−^ and H_2_O_2_. Various factors, including the specific surface area and nitrogen defective sites, are systemically studied and discussed in terms of how they influence the overall catalytic activity. A clear enhancement in organic pollutant degradation is observed using this novel system.

## 2. Experimental

### 2.1. Materials and reagents

All chemical reagents were purchased with analytical purity and used without any further purification. Deionized water (resistivity 18.2 MΩ) was filtered using a Millipore Milli-Q water purification system.

### 2.2. Preparation of O/g-C_3_N_4_ catalyst

The synthesis of the O/g-C_3_N_4_ catalyst has been reported previously by our group.^34^ Specifically, melamine powder was placed in a crucible with a cover, and then calcinated in a static air atmosphere at 550 °C for 4 h. The obtained yellow sample was denoted as g-C_3_N_4_. This g-C_3_N_4_ was added to deionized water, then transferred into a Teflon-sealed autoclave and maintained at 180 °C for 4 h. After cooling to room temperature, the obtained sample was washed with more deionized water and dried. Finally, the dried sample was placed in a crucible with a cover and calcinated for 4 h at different temperatures (350, 450, and 550 °C) in a static air atmosphere. The resulting samples were denoted as O/g-C_3_N_4_-*T* (where *T* = 350, 450, or 550 °C). Unless otherwise stated, the sample at 550 °C was denoted as simply, O/g-C_3_N_4_. Additionally, the Na–O/g-C_3_N_4_ variant was synthesized using a wet impregnation method. Essentially, the O/g-C_3_N_4_ catalyst was added to a solution containing NaHCO_3_, which was stirred vigorously for 24 h at room temperature. Next, they were dried. Finally, the sample was placed in a crucible with a cover, and calcinated for 4 h at 300 °C in a static air atmosphere (the theory value of Na^+^ is 10 wt%).

### 2.3. Characterization

X-ray diffraction (XRD) spectroscopy was carried out with a Bruker D8-Advance X-ray diffraction instrument; N_2_-physisorption was conducted on a Quantachrome Autosorb-1 instrument at liquid-N_2_ temperature; scanning electron microscopy (SEM) was carried out on a JEOL JSM 6700 F operating at an accelerating voltage of 10 kV; transmission electron microscope (TEM) was conducted on TALOS F200 X instrument operating at an accelerating voltage of 200 kV; electron paramagnetic resonance (EPR) was conducted on Bruker A300; X-ray photoelectron spectra (XPS) was examined on Thermo Fisher Scientific using Al *Kα*; zeta potential was carried out on Zetasizer Nano ZSP instrument.

### 2.4. Degradation of organic pollutants in the dark and under illumination

The degradation reactions were performed in a flask at 25 °C, at atmospheric pressure. The reaction solution contained a certain amount of catalyst, NaHCO_3_, H_2_O_2_, and organic pollutants. The reaction mixture was stirred vigorously in the dark unless otherwise stated. For the photocatalytic reactions, the reaction mixture was placed under LED illumination, using all other reaction conditions identical to the experiments conducted in the dark (*i.e.*, same catalyst dosages and concentrations of H_2_O_2_ and HCO_3_^−^). In all cases, a certain aliquot of each reaction solution was extracted at fixed intervals and then separated. The liquid was collected, and they were analyzed using a UV-vis spectrophotometer to quantify the concentration of residual organic pollutants.

## 3. Results and discussion

### 3.1. Characterization

**Fig. 1:**
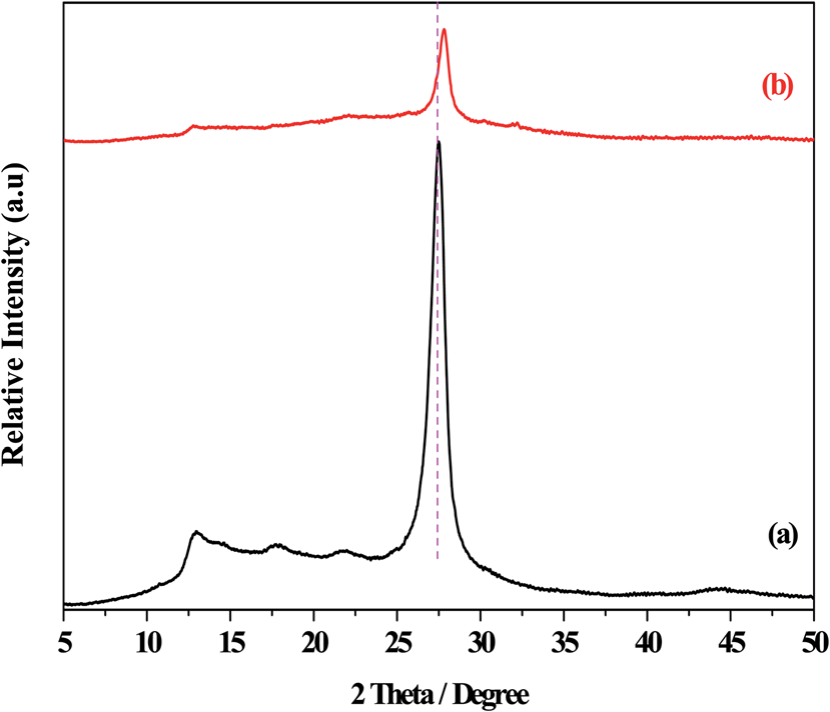
The XRD patterns of different catalysts. (a) g-C_3_N_4_, (b) O/g-C_3_N_4_.

Fig. 1 displayed the XRD patterns of the g-C_3_N_4_ and O/g-C_3_N_4_ catalysts. The diffraction peaks at 2*θ* = 13.0° and 27.5° were observed for the g-C_3_N_4_ species, and these were assigned to the repeating tri-*s*-triazine units within the g-C_3_N_4_ unit layer (100), and the inter-planar stacking of unit layers (002),^32^ respectively. Besides the characteristic peaks associated with g-C_3_N_4_, the O/g-C_3_N_4_ catalyst didn't exhibit any diffraction peaks corresponding to other phases, indicating that the original structure of g-C_3_N_4_ was largely retained following the oxygen modification. However, the strongest diffraction peaks for the O/g-C_3_N_4_ catalyst shifted toward higher angles (*i*.*e*., toward 2*θ* = 27.8°), relative to those observed for g-C_3_N_4_, representing the decreased distances within the layered structure.^11^ This change was attributed to the doping effect of oxygen atoms and the resulting distortion of the graphite structure.

The images of the g-C_3_N_4_ and O/g-C_3_N_4_ catalysts captured using SEM characterization were shown in Fig. S1.† It is clear from these images that the g-C_3_N_4_ catalyst contained agglomerated particles, and its surface appeared relatively rough. Compared to the bulk g-C_3_N_4_, a cotton-like morphology was visible in the O/g-C_3_N_4_ image. This is likely because the nanosheets of g-C_3_N_4_ were partially decomposed following the hydrothermal-calcination treatment. This difference was further confirmed through characterization using TEM characterization (Fig. S2†). Table S1† listed the contents of carbon, nitrogen, and oxygen atoms, which were determined by elemental analysis of the g-C_3_N_4_ and O/g-C_3_N_4_ samples from SEM characterization. It is observed that O/g-C_3_N_4_ contained lower nitrogen atom content but a higher atomic ratio of carbon to nitrogen, relative to g-C_3_N_4_. These results indicated that delamination and depolymerization processes were happened, causing loss of nitrogen atoms and creation of nitrogen defect sites. To confirm the existence of such defect sites, EPR measurements were carried out, and the results were presented in Fig. S3.† There were six large positive peaks observed in the spectrum of the g-C_3_N_4_ catalyst, but only four positive peaks in the O/g-C_3_N_4_ catalyst's spectrum. These results suggested that the latter had a greater quantity of unpaired electrons than the former. This can be justified based on the fact that some nitrogen atoms have been removed and other nitrogen atoms were replaced by oxygen atoms, in agreement with the XRD and elemental analysis. The analysis of all of these results led to the conclusion that the nitrogen content, and especially the morphology, of O/g-C_3_N_4_ was altered relative to those of g-C_3_N_4_.

The textural properties of g-C_3_N_4_ and the series of O/g-C_3_N_4_-*T* catalysts, which were determined from N_2_ adsorption–desorption experiments, were compiled in Table 1. Relative to the g-C_3_N_4_, and within the series of O/g-C_3_N_4_-*T* catalysts, the specific surface area (*S*_BET_), first slightly increased and then increased remarkably, up to >23 times. Simultaneously, the total pore volume (*V*_total_) first increased, and then significantly increased, up to approximately 10 times. It is well-known that g-C_3_N_4_ produced from various precursors can be easily obtained *via* high-temperature calcination, but the resulting *S*_BET_ was typically below 10 m^2^ g^−1^. This is because the interactions between the layers of g-C_3_N_4_ were too strong, owing to the van der Waals forces and/or hydrogen bonds in the material, which led to serious aggregation of the particles. After the hydrothermal treatment, these interactions were weakened due to the attacks from water molecules at high temperature and pressure. The resulting exposed nanosheets were further attacked by oxygen during the high-temperature calcination, thus creating a new morphology and increasing the *S*_BET_. Based on the SEM, TEM, and pore size distribution results (see Fig. S4†), we determined that the increased *S*_BET_ was mainly attributed to the change in the morphology of the catalyst. In general, the large *S*_BET_ is an important factor for strengthening catalytic performance, because this represents a greater proportion of exposed adsorption and catalytic active sites.

**Table 1:** Results of textural properties of various catalysts^a^

Catalysts	*S*_BET_ (m^2^ g^−1^)	*V*_total_ (cc g^−1^)
g-C_3_N_4_	11.5	0.0693
O/g-C_3_N_4_-350 °C	42.2	0.3056
O/g-C_3_N_4_-450 °C	50.7	0.3107
O/g-C_3_N_4_-550 °C	236.4	0.6344

a *S*_BET_ was denoted as the specific surface area; *V*_total_ was denoted as the total pore volume.

**Fig. 2:**
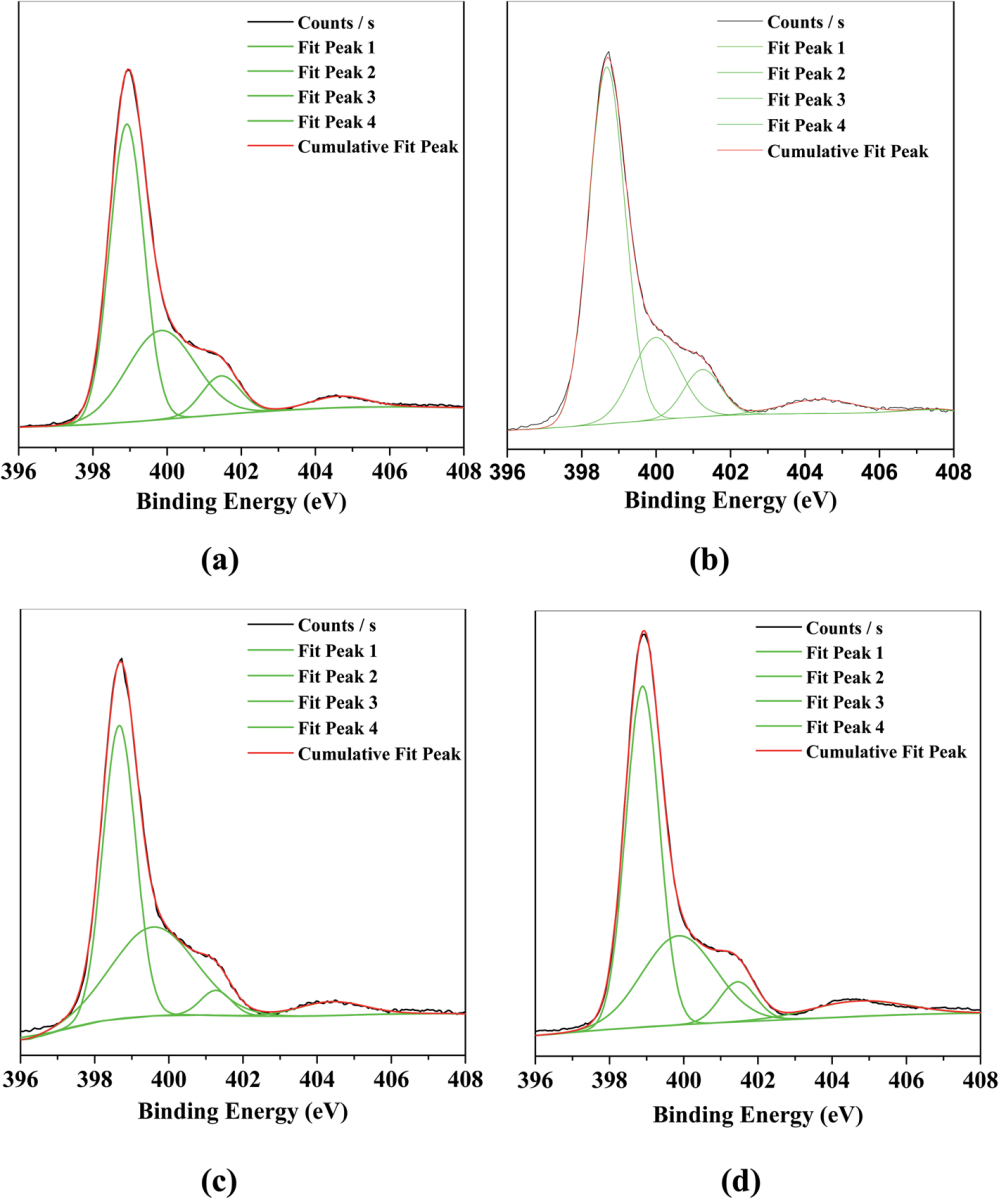
The XPS characterization for N 1s of various catalysts. (a) g-C_3_N_4_, (b) O/g-C_3_N_4_-350 °C, (c) O/g-C_3_N_4_-450 °C, (d) O/g-C_3_N_4_-550 °C.

Fig. 2 displayed the binding energies of the N 1s in g-C_3_N_4_ and the series of O/g-C_3_N_4_-*T* samples, which were determined based on XPS. In the bulk g-C_3_N_4_, four different peaks at 398.9, 399.8, 401.5, and 404.6 eV were observed, corresponding to the sp^2^-hybridized nitrogen (N–C=N; denoted as sp^2^(N)), the sp^3^-hybridized nitrogen (N–[C]_3_; denoted as sp^3^(N)), the N–H bonds, and the π band localized in heterocycles, respectively.^10,11,14–18^ The N 1s spectra associated with the series of O/g-C_3_N_4_-*T* samples contained similar peaks to the bulk g-C_3_N_4_. However, the ratio of sp^2^(N) to sp^3^(N) first increased (see Table S2†) and then decreased with increasing temperature in the O/g-C_3_N_4_-*T* series (*i.e.*, 350 °C *vs.* 450 °C *vs.* 550 °C), and as compared to g-C_3_N_4_. This result illustrated that loss of N occurred preferentially, thus forming some nitrogen defect sites.

### 3.2. Degradation of organic pollutants under different reaction conditions

#### 3.2.1. Operational parameters for organic pollutant degradation

A key process involved in enhancing the catalytic activity of O/g-C_3_N_4_ catalysts within the HCO_3_^−^/H_2_O_2_ system, was the activation of H_2_O_2_ toward ROS. To verify this concept, degradation experiments were carried out using this system (O/g-C_3_N_4_ + H_2_O_2_ ), and the results were shown in Fig. S5.† One can see that the Rhodamine B (denoted as RhB) degradation barely occurred only for H_2_O_2_ while it over the catalyst in the presence of H_2_O_2_ was completely degraded after 160 h. A low catalyst loading parameter was selected to exclude the influence of adsorption (no more than 5%), which further allowed the conclusion that the synergistic effect between O/g-C_3_N_4_ and H_2_O_2_ induced significant removal of RhB. This result suggested that some ROS were indeed generated, and these species acted to eliminate organic pollutants. In other words, O/g-C_3_N_4_ contained catalytic active sites, such as nitrogen defective sites, which promoted the activation of H_2_O_2_ toward ROS. However, the degradation rate of RhB was extremely slow under the experimental conditions. Therefore, a new strategy was implemented, in which HCO_3_^−^ was introduced into the system containing O/g-C_3_N_4_ and H_2_O_2_. The important reaction conditions were investigated in detail, and the relevant results were presented here.

##### (i) Effect of the HCO_3_^−^ concentration

**Fig. 3:**
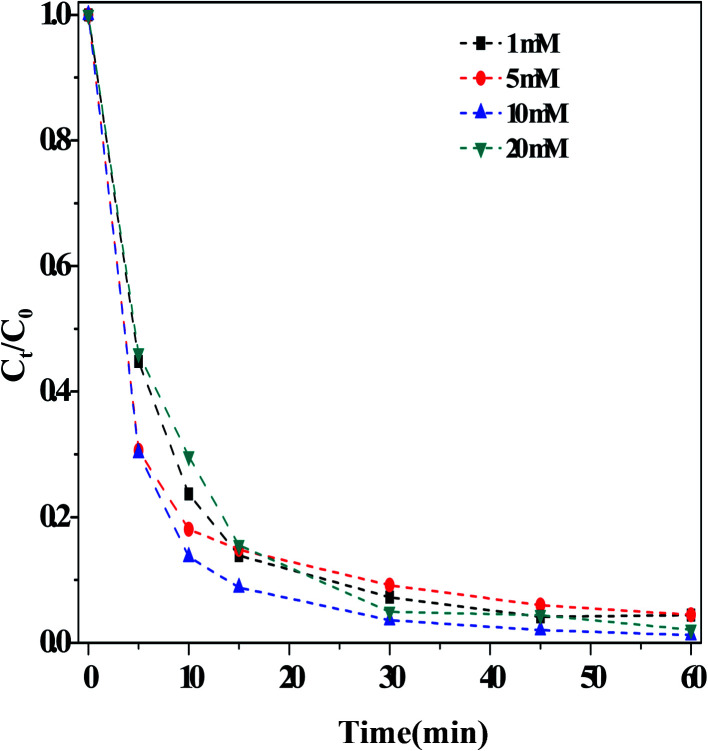
Effect of the concentrations of HCO_3_^−^ on the RhB degradation. Reaction conditions: [H_2_O_2_] = 36 mM, [O/g-C_3_N_4_] = 1.0 g L^−1^, [RhB] = 10 mg L^−1^, 25 °C and 60 min.

Fig. 3 illustrated the influence of the concentration of HCO_3_^−^ on the outcome of the dark Fenton-like reaction. It is determined that the rate of RhB degradation in the reaction solution accelerated significantly following the addition of HCO_3_^−^. Moreover, the RhB degradation rate increased with increasing concentrations of HCO_3_^−^, achieving the fastest value with 10.0 mM HCO_3_^−^. Upon increasing the concentration of HCO_3_^−^ even further, the degradation rate was reduced, although it remained prominently higher than that measured in the absence of HCO_3_^−^ (*i.e.*, when [HCO_3_^−^] > 10 mM, the reaction rate was still enhanced). These results showed that the concentration range for HCO_3_^−^ was very broad under these experimental conditions. Literature reports^16,19^ demonstrated that the impact of HCO_3_^−^ concentration on the organic pollutant degradation varied depending on the system. For example, Cheng *et al.*^19^ found that the degradation of acid orange II using a Cu(ii)–H_2_O_2_ system was restrained when the concentration of HCO_3_^−^ was greater than 5 mM. This indicated that another reaction may have taken place in solution, besides that involving the catalyst. Surprisingly, the solution pH was found to increase slightly (∼1.0 pH unit) after the reaction, as shown in Fig. S6.† Lei *et al.*^28^ observed the same phenomenon in their catalytic system containing a mixture of CuO–FeO and persulfate. According to eqn (1)–(6), singlet oxygen species can be obtained on the basis of pH changes after the reaction.

1 HCO_3_^−^ + ˙OH → CO_3_˙^−^ + H_2_O

2 CO_3_˙^−^ + H_2_O_2_ → HCO_3_^−^ + ˙OOH

3 ˙OOH → H^+^ + O_2_˙^−^

4 O_2_˙^−^ + ˙OH → ^1^O_2_ + OH^−^

5 O_2_˙^−^ + ˙OOH → ^1^O_2_ + OOH^−^

6 ˙OOH + ˙OOH → ^1^O_2_ + H_2_O_2_

##### (ii) Effect of the H_2_O_2_ concentration

**Fig. 4:**
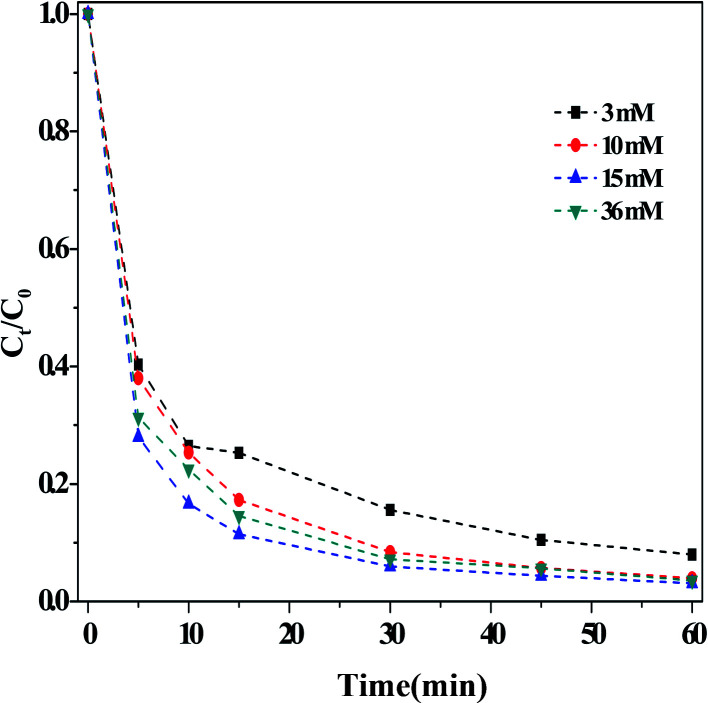
Effect of the concentration of H_2_O_2_ on the RhB degradation. Reaction conditions: [HCO_3_] = 10 mM, [O/g-C_3_N_4_] = 1.0 g L^−1^, [RhB] = 10 mg L^−1^, 25 °C, pH ≈ 8.4 and 60 min.

Fig. 4 depicted the influence of the concentration of H_2_O_2_ on the dark Fenton-like reaction. It is clear that the RhB degradation efficiency increased with increasing concentrations of H_2_O_2_, arriving at a maximum rate when [H_2_O_2_] = 15 mM. Increasing the H_2_O_2_ concentration further did not lead to any change in the efficiency of RhB degradation. As mentioned above, the ROS were generated from the decomposition of H_2_O_2_, so it followed that, when the concentration of H_2_O_2_ was relatively lower, fewer ROS could be formed. Therefore, by increasing the concentration of H_2_O_2_, the efficiency of RhB degradation was enhanced. However, the H_2_O_2_ concentration was increased further, the negligible influence on the RhB degradation indicated that the overall reaction predominately relied on the action of the catalyst.

##### (iii) Effect of the O/g-C_3_N_4_ catalyst

**Fig. 5:**
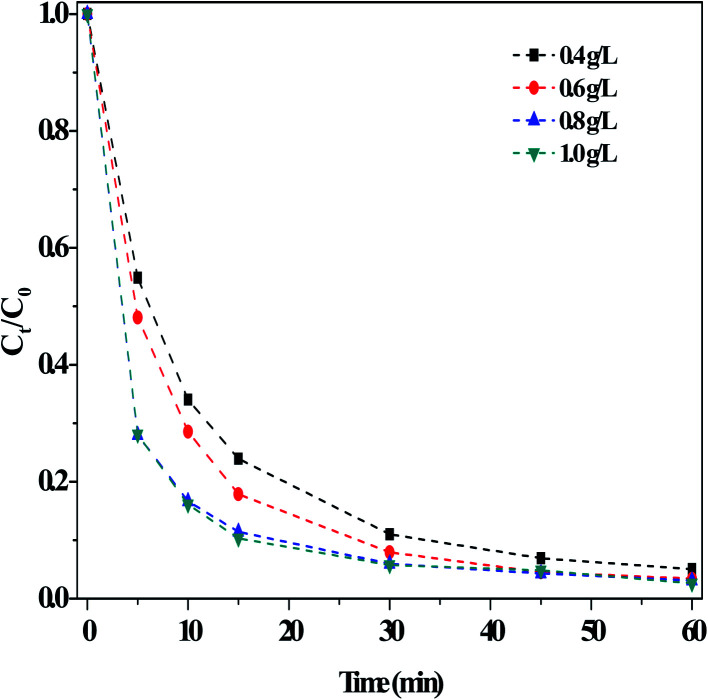
Effect of the usages of O/g-C_3_N_4_ on the RhB degradation. Reaction conditions: [NaHCO_3_] = 15 mM, [H_2_O_2_] = 10 mM, [RhB] = 10 mg L^−1^, 25 °C, pH ≈ 8.4 and 60 min.

Fig. 5 illustrated the impact of the O/g-C_3_N_4_ catalyst on RhB degradation in the dark Fenton-like reaction. The degradation of RhB clearly increased with increasing catalyst loading, reaching a maximum value at a catalyst concentration of 0.8 g L^−1^. The rate remained stable when additional catalyst was added. It is evident that the removal of RhB was closely related to the catalyst dosage, as shown in Fig. S7.† This is because a larger quantity of catalyst offered more adsorption sites, thus enabling more RhB removal. However, addition of too much catalyst led to agglomeration and increased counter forces between the particles, ultimately reducing RhB adsorption. In contrast, an insufficient amount of catalyst was introduced, there would not have enough available sites to activate H_2_O_2_ for producing ROS, so the RhB degradation rate stabilized or may decrease slightly.

Based on the described systematic screening experiments, the optimal reaction conditions were determined: [NaHCO_3_] = 10 mM, [H_2_O_2_] = 15 mM, and [O/g-C_3_N_4_] = 0.8 g L^−1^. Applying these conditions, other important parameters were investigated.

Fig. S8† showed the influence of metal ions (with Cl^−^ as the counter anion) on RhB degradation in the dark Fenton-like reaction. Based on this figure, there appeared to be no clear difference in RhB degradation among the tested metal ions, indicating that these ions had no appreciable effect on the RhB degradation reaction. This is because they were not involved the catalytic reaction, but rather, only impact solubility. For example, Ca^2+^ decreased the solubility of reaction solution, resulting in a decreased concentration of HCO_3_^−^. Many previous studies^12–19^ demonstrated that certain transition metal ions, such as Co(ii) and Cu(ii), effectively degraded organic pollutants due to the formation of M[HCO_3_^−^-H_2_O_2_]_2_RhB complexes. These complexes were formed when, for example, Co^2+^ interacted with HCO_3_^−^ to form a polymeric M^*n*+^[HCO_3_^−^] species, which then adsorbed H_2_O_2_. As a result, the ˙OH radicals were immediately generated, and attacked the RhB molecules on the surface of the catalyst. These processes led to a remarkable enhancement in RhB degradation. In contrast to transition metal ions, the metal ions studied herein were widely distributed in natural water environments, and they did not form M[HCO_3_^−^–H_2_O_2_]_2_RhB complexes. We additionally studied the influence of several anions (using Na^+^ as the metal ion), and the results were displayed in Fig. S9.† It is determined that most anions, except for CO_3_^−^ and CH_3_COO^−^, positively impacted the RhB degradation. These results demonstrated that the studied catalytic system was resilient in terms of metal ions and anions, so it represented great potential for applications in industry. The removal of other organic pollutants was also investigated, and the results were shown in Fig. S10.† All of the tested species were removed effectively using this system, further verifying that it was favorable for degrading various organic contaminants.

Fig. S11† displayed the impact of darkness *versus* LED illumination on the degradation of RhB. It is evident that the system containing O/g-C_3_N_4_, HCO_3_^−^, and H_2_O_2_ exhibited better catalytic activity under LED illumination, relative to in the dark. This is because additional light-induced reactions occurred simultaneously with the dark Fenton-like reaction, as described by eqn (7)–(9); these represented a typical photo-Fenton-like reaction process. Specifically, under LED light, the photo-generated hole (h^+^) directly reacted with RhB, and the photo-generated electron (e^−^) interacted with the dissolved oxygen in aqueous solution to form the O_2_˙^−^ species. These species could remove RhB, so the degradation of RhB was enhanced under LED illumination. The results of these experiments clearly showed that this catalytic degradation process could proceed under both light and dark conditions. This is a particularly useful finding for this new non-metal catalytic system, which is capable of eliminating organic pollutants, thus reducing environmental contamination as much as possible. On the contrary, traditional carbon-based materials that promote Fenton-like reactions doesn't happen under light illumination, so the system developed herein clearly has an advantage in its ability to function in the dark.

7 Catalyst + *hv* → h^+^ + e^−^

8
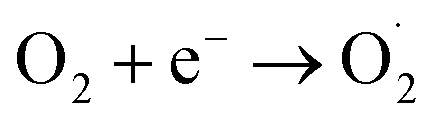

9 h^+^ (or O_2_˙^−^) + organics → CO_2_ + H_2_O

**Fig. 6:**
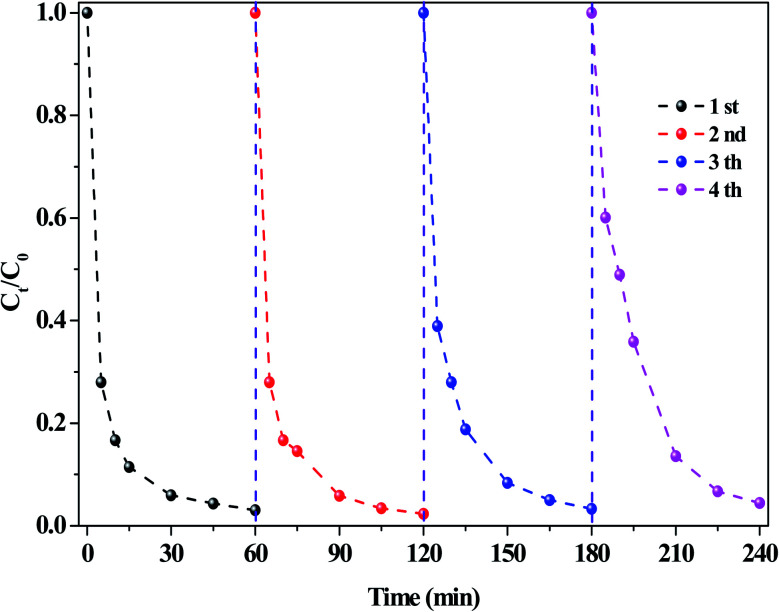
The stability of catalysts on the RhB degradation. Reaction conditions: [NaHCO_3_] = 10 mM, [H_2_O_2_] = 15 mM, [O/g-C_3_N_4_] = 0.8 g L^−1^, [RhB] = 10 mg L^−1^, 25 °C, pH ≈ 8.4 and 60 min.

The stability of the O/g-C_3_N_4_ catalyst was investigated, and the results were presented in Fig. 6. Cyclic RhB degradation experiments were conducted under dark conditions. Specifically, after the reaction, the catalyst was carefully washed with deionized water and ethanol, respectively, and then dried. Afterward, it was employed for the next cycle following the addition of a fresh reaction solution containing HCO_3_^−^ and H_2_O_2_, wherein the concentration of these components remained the same as in the first reaction. As shown in Fig. 6, the RhB degradation rate hardly decreased, even after several reaction cycles, thereby verifying the high stability of the system and repeatability of the process.

The degradation of organic dyes in wastewater was completed using the system containing HCO_3_^−^ and H_2_O_2_, and the results were compiled in Table 2. Based on these reactions, the HCO_3_^−^ and H_2_O_2_ consumptions were significant in the absence of catalyst. When Co(ii) or Cu(ii) was added into the reaction solution, significant degradation of the organic dyes was always observed. The main drawbacks associated with these catalysts included their cost, high toxicity, and difficult recovery. In order to find a suitable substitute for homogeneous catalysts, one current method involved using heterogeneous catalysts. For example, diatomite-supported cobalt,^21^ CoMgAl–Na–Y,^22^ and S/CoFe_2_O_4_ (ref. ^23^) have been applied for removing organic pollutants; however, these options also faced problems, such as their cost and complex preparation. Importantly, leaching of metal ions from these heterogeneous catalysts was inevitable and caused secondary contamination. These factors constituted the main challenges that must be overcome to allow their industrial application. Relative to such heterogeneous variants, the HCO_3_^−^–H_2_O_2_–O/g-C_3_N_4_ catalytic system was green, inexpensive, and easy to operate. These advantages confirmed that the novel technology presented herein represented high potential for practical applications in the removal of organic pollutants from wastewater.

**Table 2:** The degradation of various organic dyes over kinds of catalysts in the presence of H_2_O_2_ and NaHCO_3_

Catalysts	Organics	Reaction conditions	Degradation	Ref.
No	MB, MO, RhB	C(H_2_O_2_) = 0.1 M, C(NaHCO_3_) = 0.5 M	∼100%, 300 min	5
Co(ii)	MB	C(Co(ii)) = 20 μM, C(H_2_O_2_) = 20 mM, C(NaHCO_3_) = 25 mM	∼100%, ∼50 min	13
	AOII	C(Co(ii)) = 5 μM, C(H_2_O_2_) = 4 mM, C(NaHCO_3_) = 10 mM	>90%, 10 min	15
	X-3B		>90%, 40 min	16
Cu(ii)	AOII	C(Cu(ii)) = 0.03 mM, C(H_2_O_2_) = 4 mM, C(NaHCO_3_) = 10 mM	∼100%, 10 min	19
Diatomite-supported cobalt	MB	C(Catal.) = 0.4 g L^−1^, C(H_2_O_2_) = 60 mM, C(NaHCO_3_) = 25 mM	∼99%, 80 min	21
	RhB		70%, 5 h	
CoMgAl–Na–Y	MO	C(Catal.) = 0.6 g L^−1^, C(H_2_O_2_) = 50 mM, C(NaHCO_3_) = 25 mM	100%, 10–60 min	22
	MB^a^	F(Catal.) = 3 mL g^−1^ h^−1^, C(H_2_O_2_) = 25 mM, C(NaHCO_3_) = 48 mM	100%, 0–132 h; 96–98%, 132–312 h	
S/CoFe_2_O_4_	AOII	C(Catal.) = 0.1 g L^−1^, C(H_2_O_2_) = 3 mM, C(NaHCO_3_) = 0.1 g L^−1^	∼99%, 15 min	23
O/g-C_3_N_4_	RhB	C(Catal.) = 0.8 g L^−1^, C(H_2_O_2_) = 15 mM, C(NaHCO_3_) = 10 mM	∼90%, 15 min	In this work
	MB		>90%, 5 min	
	RhB^b^	C(Catal.) = 0.1 g L^−1^, C(H_2_O_2_) = 15 mM, C(NaHCO_3_) = 10 mM	>90%, 30 min	

a A fixed-bed reactor.

b LED illumination.

#### 3.2.2. The mechanism of organic pollutant removal

To identify whether various ROS were produced in the system containing HCO_3_^−^ and H_2_O_2_ with the O/g-C_3_N_4_ catalyst, we attempted to detect selected ROS, including ˙OH, O_2_˙^−^, and ^1^O_2_, using different radical scavengers.^21^ First, a common radical scavenger for ˙OH, ascorbic acid, was introduced into the reaction mixture solution. As observed in Fig. S12,† addition of ascorbic acid caused the degradation rate to decrease. However, it did not completely quench the reaction, suggesting the existence of a radical-mediated degradation pathway, except for the adsorption process. Ethanol was a relatively more powerful radical scavenger, and Fig. S13† displayed the influence of ethanol on RhB degradation. It is clear that the addition of ethanol significantly decreased RhB degradation, and this inhibition effect was concentration-dependent over a wide range. Therefore, we concluded that the ˙OH species was involved this reaction, but it was not the only ROS.

In order to probe the production of O_2_˙^−^ along the reaction pathway, the influence of different concentrations of the scavenger, Trion, on the RhB degradation was investigated. It is clear from Fig. S14† that the RhB degradation in the presence of 5 mM Trion decreased remarkably at first and then stabilized with increasing reaction time. This trend was also observed at higher concentrations (50 and 200 mM) of Trion. These results suggested that the O_2_˙^−^ radical was an important ROS. Another radical scavenger, benzoquinone, was also studied in this reaction, and the results were shown in Fig. S15.† It is determined that the degradation of RhB decreased with the addition of 3 and 6 mM of benzoquinone, respectively. This confirmed that the O_2_˙^−^ radical had an important role in the removal of organic pollutants in this system.

Fig. S16†shows the effect of different concentrations of NaN_3_ (used for singlet oxygen detection) on the RhB degradation. In this case, the change toward the RhB degradation was not significance at two different NaN_3_ concentrations (4 and 10 mM). Therefore, we determined that ^1^O_2_ was not a major ROS in this degradation mechanism. In order to support this conclusion, another experiment was carried out by adding furfuryl alcohol (FFA), rather than NaN_3_, into the reaction, and the results were shown in Fig. S17.† Under these conditions, the RhB degradation decreased only slightly relative to that of the control experiment. This fact confirmed that ^1^O_2_ was not a primary participant in this reaction, and it was consistent with the analysis of Fig. S6.† Overall, the mechanistic analysis indicated that ˙OH and O_2_˙^−^ radicals were important ROS for this reactivity, but the ^1^O_2_ species was little involved.

#### 3.2.3. The synergistic effect between adsorption and chemical oxidation

**Fig. 7:**
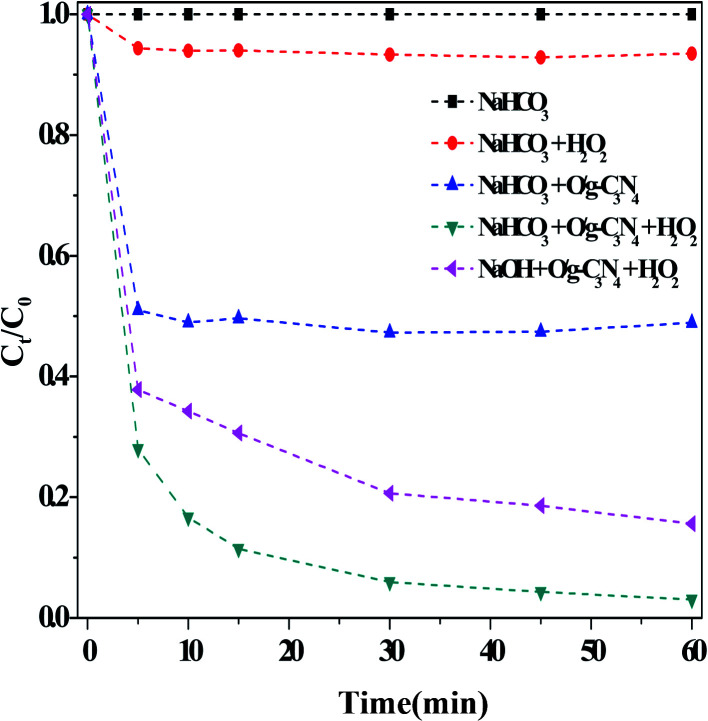
Comparison results of different catalysts on the RhB degradation. Reaction conditions: [NaHCO_3_] = 10 mM, [H_2_O_2_] = 15 mM, [O/g-C_3_N_4_] = 0.8 g L^−1^, [RhB] = 10 mg L^−1^, 25 °C, pH ≈ 8.4 and 60 min.

To fully understand the contributions of the adsorption process and ROS production, several important factors were investigated under the dark conditions, and the results were presented in Fig. 7. It is clear that HCO_3_^−^ alone was hardly capable of degrading RhB, and the degradation of RhB was extremely low even after treating with a 10 mM HCO_3_^−^ solution in the presence of H_2_O_2_. This finding contradicted several previous reports,^2–11^ likely due to the different types of organic pollutants used in the experiments and the conditions of use for HCO_3_^−^ and H_2_O_2_. For examples, in previous studies,^5,8–11^ the organic pollutants were directly degraded by activating H_2_O_2_, which required a high concentration of HCO_3_^−^ (*i.e.*, 500 mM). In general, these systems exhibited lower degradation if small HCO_3_^−^ and H_2_O_2_ concentrations were incorporated; however, Kan *et al.*^11^ discovered that the acid orange II was effectively degraded using low concentrations of HCO_3_^−^ and H_2_O_2_. According to the literature,^25^ [HCO_4_^−^]_*e*_ = *K*_eq_{[HCO_3_^−^]_0_·[H_2_O_2_]_0_}/{1 + *K*_eq_[HCO_3_^−^]_0_}, where *K*_eq_ = 0.33 M^−1^ at 25 °C, and the calculated [HCO_4_^−^]_e_ was approximately 49 μM. This concentration was about two times that of RhB, and this anion had strong oxidizing ability,^3,8,21^ despite a slow reaction rate. This indicated that the HCO_4_^−^ species was not active in this reaction (eqn (10)), consistent with literature precedent.^19^

10 HCO_3_^−^ + H_2_O_2_ ↔ HCO_4_^−^ + H_2_O

The activated pathway, comprised of HCO_3_^−^ + H_2_O_2_ + O/g-C_3_N_4_, demonstrated an increased degradation efficiency (>90% after 60 min). As reported previously,^37^ the electrons near nitrogen defect sites could elongate the O–O bonds in H_2_O_2_ molecules, thereby reducing the activation energy. We have already demonstrated that the O/g-C_3_N_4_ catalyst contained many such defect sites, which could directly activate H_2_O_2_ in the dark (Fig. S5†). As a result, some ROS, including ˙OH radicals, were generated and served to enhance RhB degradation.^34^ Similarly, the addition of NaOH rather than NaHCO_3_ into the reaction solution generated a similar pH environment (∼8.4) and enhanced the degradation of RhB, although not as much as HCO_3_^−^. In addition, the ∼50% enhancement in degradation efficiency over O/g-C_3_N_4_ was obtained due to HCO_3_^−^, and this increase was mainly attributed to the adsorption aspect.

Overall, there are two main reasons for the enhancement exhibited by this system. The first involved the extremely high specific surface area of the O/g-C_3_N_4_ catalyst, which was beneficial for improving the removal of RhB *via* an adsorption process. In theory, the p*K*_a_ of g-C_3_N_4_ obtained from melamine was about 3,^38^ meaning that it could easily combine with RhB through electrostatic interactions (RhB was a positively charged molecule, and the surface of g-C_3_N_4_ presented a negative charge). The zeta potential of the catalyst was determined at pH ≈ 8.4, and this value was about −19 mV. In general, the specific surface area of the catalyst was high, so its adsorption capacity for RhB was large; consequently, the enhanced degradation was attributed partially to the high specific surface area of the catalyst. The second reason for the enhanced activity of the developed catalytic system involved ROS production. In advanced oxidation technologies, ˙OH radicals are well-known catalytic active species for the degradation of various organic pollutants, with redox potentials in acidic and alkaline conditions of +2.8 V and +1.5 V, respectively.^21^ The NaOH or NaHCO_3_ environment provided a weak alkaline medium and also served to activate H_2_O_2_, thus inducing the formation of more ROS. However, some ROS were produced as a result of the synergistic behavior among NaHCO_3_, H_2_O_2_, and O/g-C_3_N_4_. As previously stated,^21^ the CO_3_˙^−^ radicals were formed by the reaction shown as eqn (1), and these radicals may be even more effective for the degradation of organic dye molecules than ˙OH radicals. The HCO_3_^−^ anion was often employed as scavenger to detect whether ˙OH radicals were presented in the reaction solution. However, it has been reported that HCO_3_^−^ could accelerate the degradation of organic pollutants under certain reaction conditions, *i.e*., with specific catalysts or at specified concentrations.^8,11–27^ This is because the ˙OH radicals (i) had shorter lifetimes due to the potential self-combination process,^32^ and (ii) could react with H_2_O_2_ to produce O_2_.^32^ In contrast, CO_3_˙^−^ radicals had longer lifetimes due to coulombic repulsion.^21^ In fact, it has been demonstrated that CO_3_˙^−^ radicals could accelerate the degradation of organic pollutants,^21^ or generate other effective radicals (*e.g.*, ˙OOH, O_2_˙^−^ and ^1^O_2_) that were active for organic pollutant degradation under different conditions (eqn (2)–(6)).^21^

In this work, the ˙OH radicals were first generated by activating H_2_O_2_ at the defective sites on the O/g-C_3_N_4_ catalyst. Some ˙OH radicals left the surface of the catalyst and entered the reaction solution. As mentioned above, some of these ˙OH radicals immediately reacted with each other, thus contributing to their extremely short measured lifetimes. However, if the reaction solution contained an adequate concentration of HCO_3_^−^ ions, the ˙OH radicals may react with HCO_3_^−^ ions to form CO_3_˙^−^ radicals. In this case, the RhB degradation continued, and the pollutant removal efficiency increased further. This phenomenon was evidenced by the relationship between HCO_3_^−^ concentration and RhB degradation, as illustrated in Fig. 3. Based on eqn (1), the formation of CO_3_˙^−^ radicals was mainly due to the reaction between HCO_3_^−^ and ˙OH. Therefore, the increased concentrations of HCO_3_^−^ theoretically led to increased quantities of CO_3_˙^−^ radicals, thus improving RhB degradation. However, if the concentration of HCO_3_^−^ is too high, more CO_3_˙^−^ and related radicals were generated (eqn (2)–(6)), resulting in a remarkable reduction in the concentration of ˙OH radicals. These conditions led to a lesser degree of RhB degradation, and clearly, this phenomenon was not observed in the NaOH-containing system.

Overall, the optimal RhB degradation efficiency was obtained using the HCO_3_^−^–H_2_O_2_–O/g-C_3_N_4_ system. In this case, ROS, including ˙OH, O_2_˙^−^, ^1^O_2_, and CO_3_˙^−^, attacked the organic pollutants through a chemical process, leading to the destruction of the RhB structure. The structural change was further confirmed based on UV-vis results, as shown in Fig. S18.† It is worth noting that there was a large difference in the Na^+^ content in the NaOH and NaHCO_3_ systems (negligible (μM) *vs.* 10 mM, respectively), and its role was not thoroughly taken into consideration. To certify this assumption, preliminary experiments were conducted using Na–O/g-C_3_N_4_ as the catalyst, and the results were displayed in Fig. S19,† clearly showing that there was little influence on the removal of RhB.

**Scheme 1:**
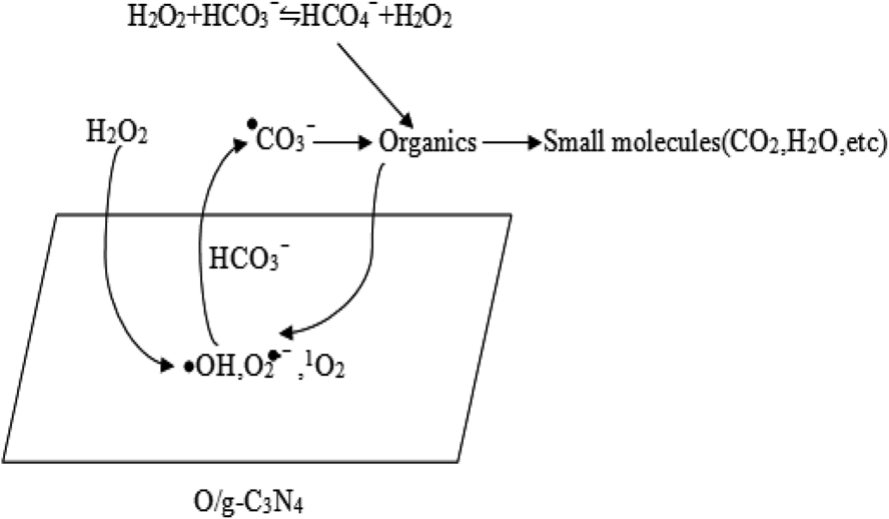
The degradation pathway of organic pollutants.

In summary, the high degradation capability of the HCO_3_^−^–H_2_O_2_–O/g-C_3_N_4_ system was mainly ascribed to the synergistic effect between adsorption and chemical oxidation. The HCO_3_^−^ and O/g-C_3_N_4_ components were responsible for activating H_2_O_2_ and removing RhB. Specifically, the O/g-C_3_N_4_ catalyst acted as a bifunctional material for dispelling RhB, thus contributing to the simultaneous adsorption and chemical oxidation processes. The huge specific surface area of the O/g-C_3_N_4_ catalyst allowed significant adsorption *via* a non-radical pathway, but the chemical oxidation was carried out through a radical route, which relied on the defective sites of O/g-C_3_N_4_ for activating H_2_O_2_ to ROS in the presence of HCO_3_^−^. Chemical oxidation also occurred in the reaction solution itself, and this mechanistic pathway was outlined in Scheme 1.

#### 3.2.4. The relationship between the catalyst's structure and its catalytic activity

To firmly establish the relationship between the structure of the catalyst and its ability to degrade RhB, the effect of the calcination temperature when preparing O/g-C_3_N_4_-*T* was investigated. It is known that the calcination temperature influenced the specific surface area and defective sites of the resulting O/g-C_3_N_4_. Fig. S20† illustrated the effect of the calcination temperature across the series of O/g-C_3_N_4_-*T* catalysts on their RhB degradation performances. The O/g-C_3_N_4_-550 catalyst exhibited the maximum RhB degradation efficiency among all variants tested under identical conditions; the catalysts prepared with lower calcination temperatures demonstrated less RhB degradation efficiency (especially below 450 °C). This behavior was likely due to their relatively lower specific surface areas and intact polymerization. Additionally, a higher calcination temperature induced a tremendous change to the g-C_3_N_4_ structure, particularly at 550 °C. In general, the higher the calcination temperature is employed, the greater the separation between nanosheets of g-C_3_N_4_ is happened. It can also easily decompose to generate small gaseous products, which diffuse between the layers of nanosheets. In this case, the morphology of the catalyst is greatly altered, leading to a higher specific surface area, as confirmed by SEM and TEM characterizations. Simultaneously, numerous defect sites, such as vacancies and edges, are generated. The resulting increased specific surface area is favorable for molecular adsorption, because it offers more catalytic active sites (the defect sites can activate H_2_O_2_ toward ROS *in situ*).^39–44^ However, no interesting products are obtained if the calcination temperature is too high (>550 °C). To test this hypothesis, the relationships between the S_BET_, the content and ratio of N(sp^2^) and N(sp^3^) (denoted as defect sites), and the catalytic activity were evaluated, and the results were compiled in Table 1 and S2.† The S_BET_ increased gradually with applied temperatures below 550 °C and then significantly improved at 550 °C, clearly indicating that the *S*_BET_ was closely related to the catalytic activity. Accordingly, increasing the *S*_BET_ of the catalyst led to increased RhB degradation. Meanwhile, the N(sp^2^) and N(sp^3^) ratio first increased, when changing from 350 °C to 450 °C, but then began to decrease at 550 °C. This behavior suggested that the defect sites corresponding to N(sp^3^) can to some extent act as catalytic active sites. Combined with the analysis of the degradation efficiency, we concluded that the *S*_BET_ and the defective sites from N(sp^3^) were two important factors for enhancing the catalytic activity of the developed system.

## 4. Conclusions

This report discussed a newly-developed strategy for treating wastewater polluted with organic contaminants. The employed system consisted of HCO_3_^−^, H_2_O_2_, and an O/g-C_3_N_4_ catalyst, which together demonstrated efficient degradation of organic compounds. Various influencing factors were investigated, and the reaction conditions were optimized in terms of the catalyst loading and preparation, concentrations of HCO_3_^−^ and H_2_O_2_, and light *vs.* dark operation. The catalytic system retained high stability and exhibited sufficient reproducibility after running several reaction cycles. Mechanistic probing revealed that the degradation pathway was dominated by the synergistic effect between adsorption and chemical oxidation processes. The adsorption was closely related to the tunable specific surface area of the catalyst, and the chemical oxidation was achieved by various ROS. Overall, this work presented a novel, widely-applicable method for removing organic pollutants from wastewater.

## Conflicts of interest

There are no conflicts to declare.

## Supplementary Material

RA-011-D0RA07893J-s001
