# Supplementary material for: Oxygen doped graphitic carbon nitride nanosheets for the degradation of organic pollutants by activating hydrogen peroxide in the presence of bicarbonate in the dark
Source: RSC Adv. 2020 Dec 24;11(1):296–306. doi: 10.1039/d0ra07893j (PMC8691115; doi:10.1039/d0ra07893j)
Supplement: RA-011-D0RA07893J-s001 [file RA-011-D0RA07893J-s001.pdf]

Electronic Supplementary Material (ESI) for RSC Advances.
This journal is © The Royal Society of Chemistry 2020

Support Materials

Oxygen Doped Graphitic Carbon Nitride Nanosheets for the Degradation of Organic Pollutants by Activating Hydrogen Peroxide in the Presence of Bicarbonate in the Dark

Tian-Jiao Jiang [a,1], Chao Xie [a,1], Huai-De Peng [a], Bo Lei [a], Qing-Qing Chen [a], Gang Li [a], Cai-Wu Luo [a, b, c, d, *]

[a]School of Resource Environmental and Safety Engineering, University of South China 421000, China

[b]State Key Laboratory of Safety and Health for Metal Mines, Sinosteel Maanshan General Institute of Mining Research Co., Ltd 243000, China

[c]Key Laboratory of Clean Energy Material, LongYan University 364012, China

[d]Research Center for Eco-Environmental Sciences, Chinese Academy of Sciences 100085, China

[1] They contributed equally to this work;

* Corresponding author: Cai-Wu Luo.

Tel: +86-734-8282345;

E-mail addresses: luocaiwu00@126.com.

Figure S1

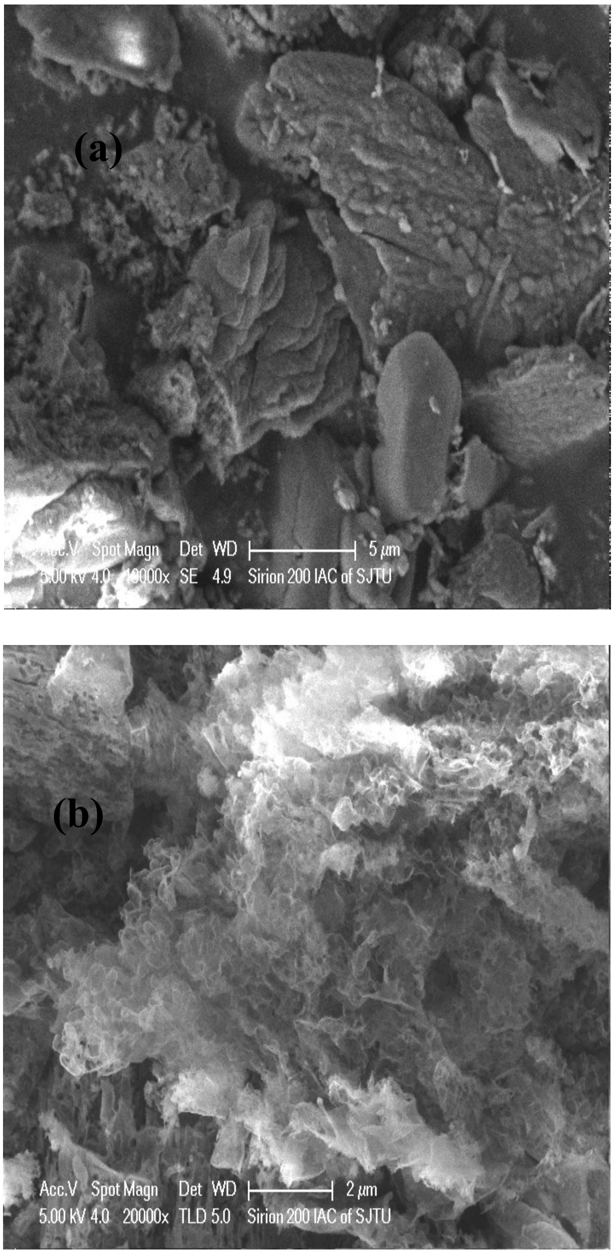

Figure S1 The SEM images of different catalysts.

(a) g-C_3N_4, (b) O/g-C_3N_4.

Figure S2

(a)

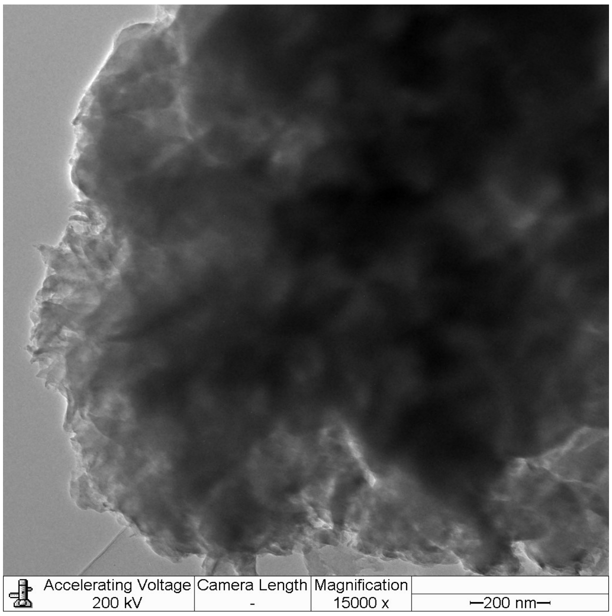

(b)

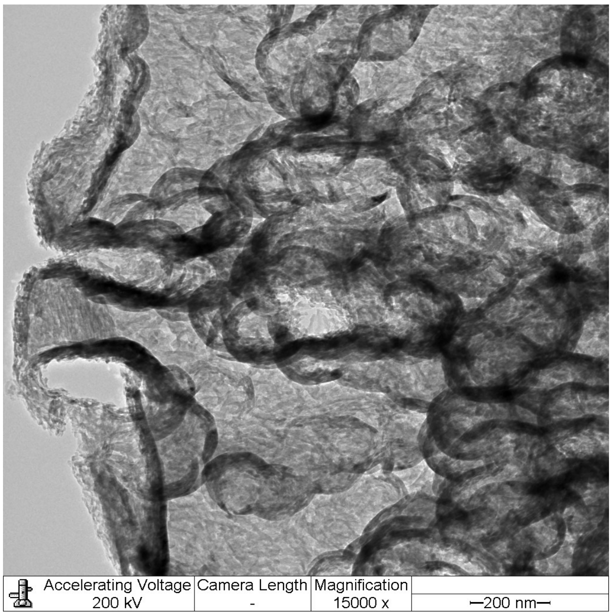

(b) Figure S2 The TEM images of different catalysts.

(a) g-C_3N_4, (b) O/g-C_3N_4.

Figure S3

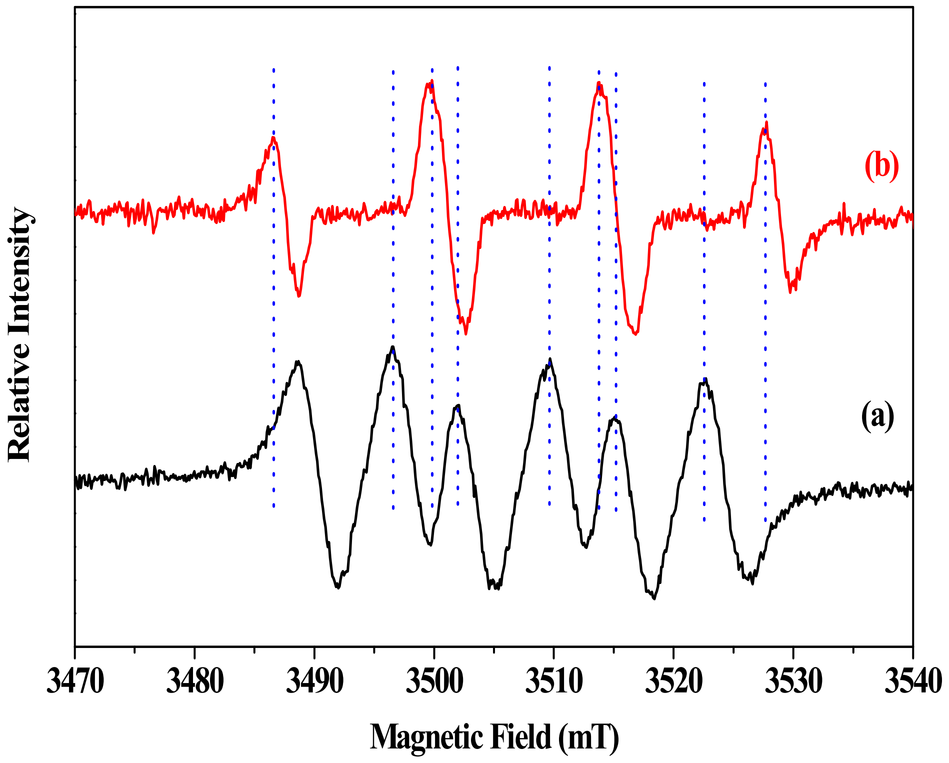

Figure S3 The EPR spectra of different catalysts.

(a) g-C_3N_4, (b) O/g-C_3N_4.

Figure S4

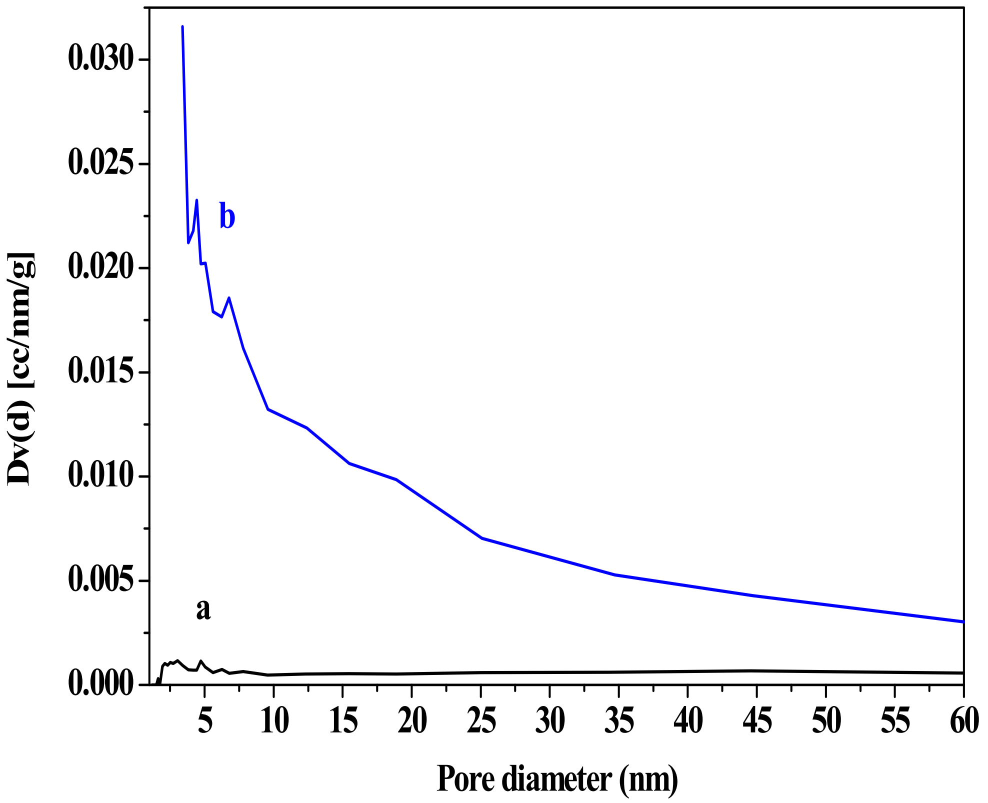

Figure S4 The pore size distributions of different catalysts (BJH method).

(a) g-C_3N_4, (b) O/g-C_3N_4.

Figure S5

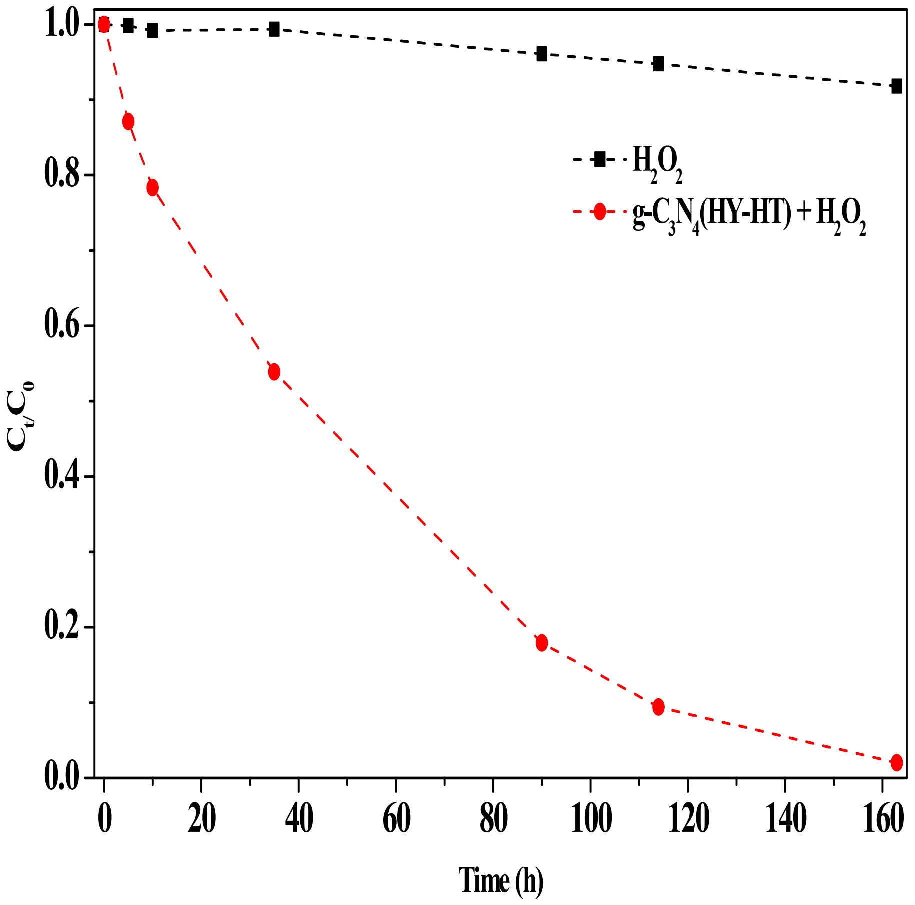

Figure S5 Effect of reaction time on the removal of RhB in the dark. Conditions: $[RhB]_0$ = 10 mg L^{-1}, $[H_2O_2]$ = 36 mM, [Catal.] = 0.1 g L^{-1}, initial pH no adjustment and 25 ^oC.

Figure S6

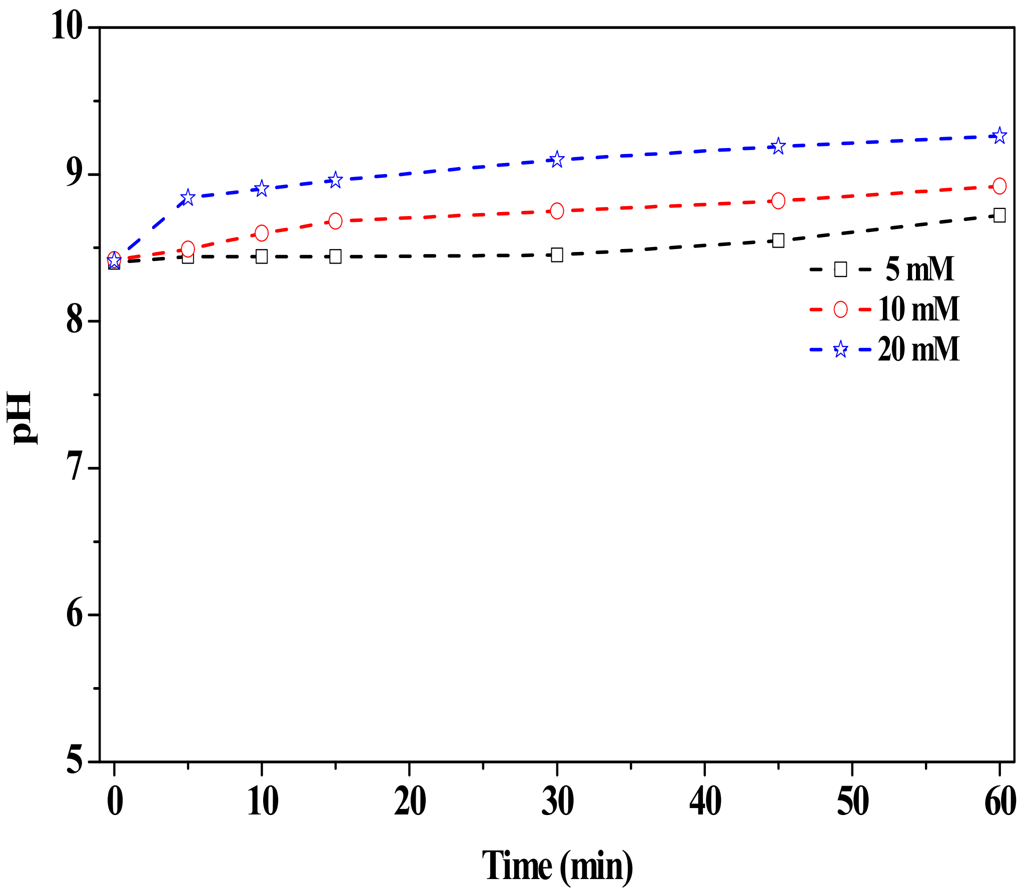

Figure S6 The change of solution pH with HCO_3^- concentration during reaction. Reaction conditions: $[H_2O_2]$ = 36 mM, $[O/g\text{-}C_3N_4]$ = 1.0 g L^{-1}, [RhB] = 10 mg L^{-1}, 25°C and 60 min.

Figure S7

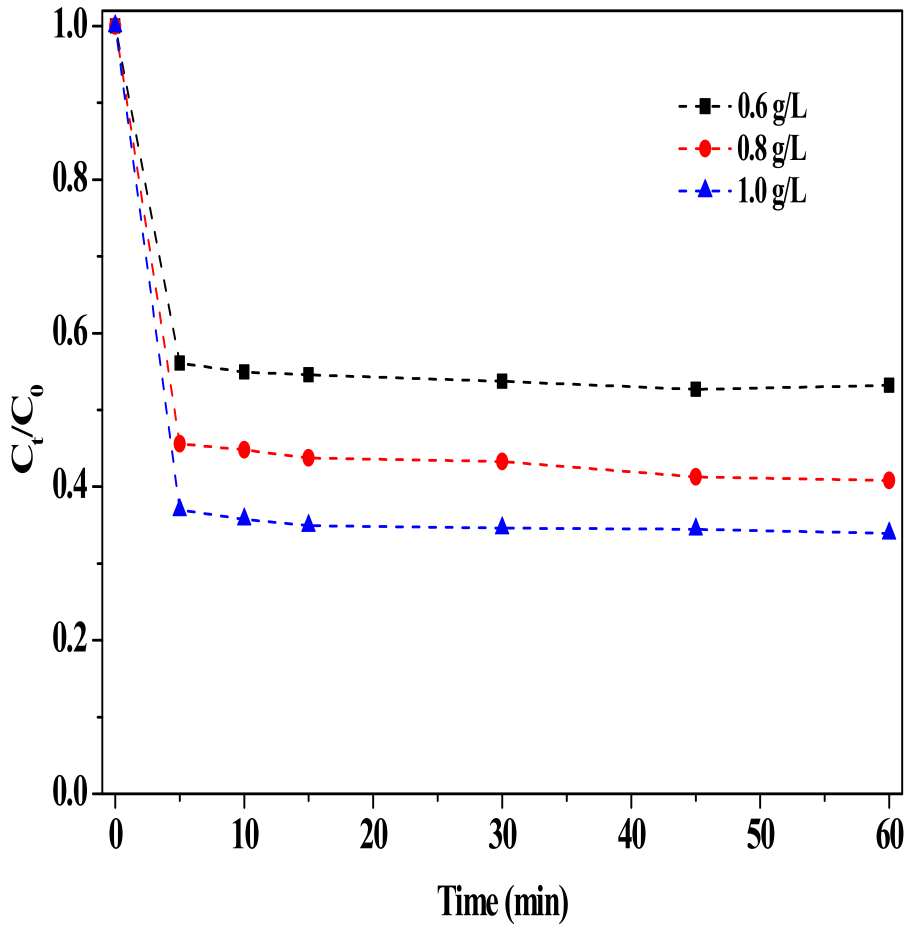

Figure S7 The effect of the mass of catalyst on the removal of RhB through the absorption. Reaction conditions: [RhB] = 10 mg L^{-1}, 25°C and 60 min, [O/g-C_3N_4] = 0.6 g L^{-1}, 0.8 g L^{-1} and 1.0 g L^{-1}, respectively.

Figure S8

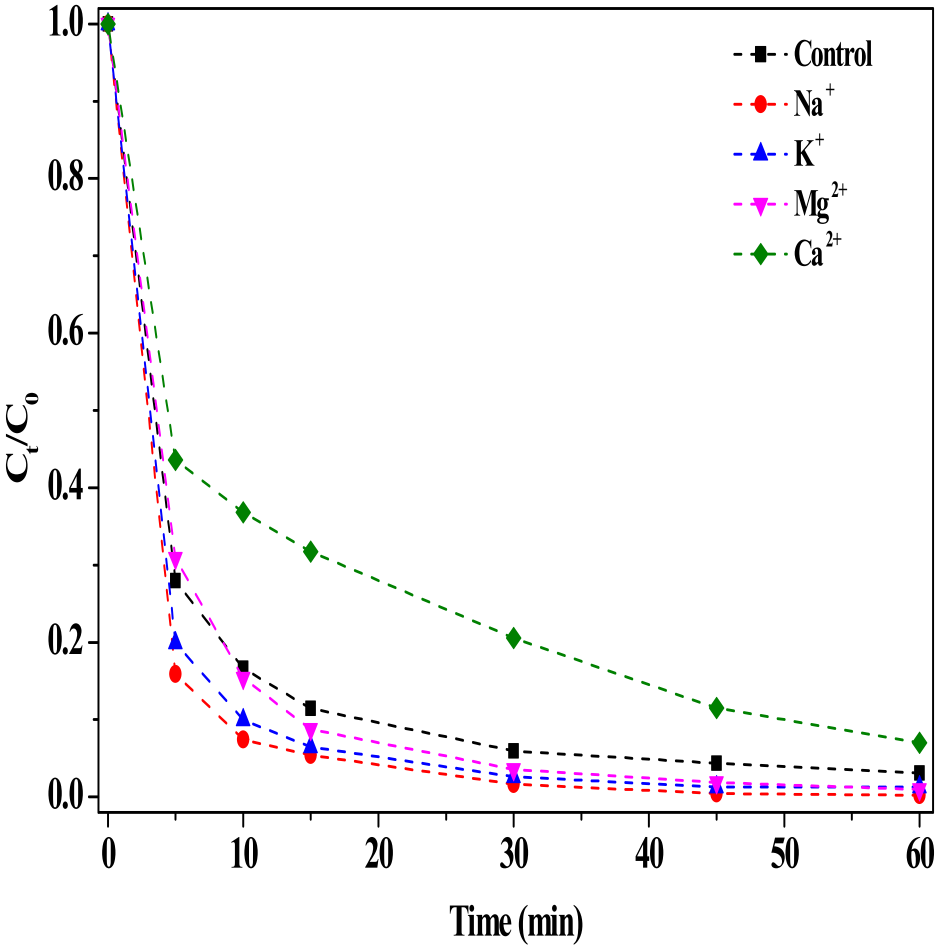

Figure S8 Effect of various metal ions on the RhB degradation. Reaction conditions: $[NaHCO_3]$ = 10 mM, $[H_2O_2]$ = 15 mM, $[O/g\text{-}C_3N_4]$ = 0.8 g L^{-1}, [Metal ions] = 100 mM, [RhB] = 10 mg L^{-1}, 25 ^oC, pH ≈ 8.4 and 60 min.

Figure S9

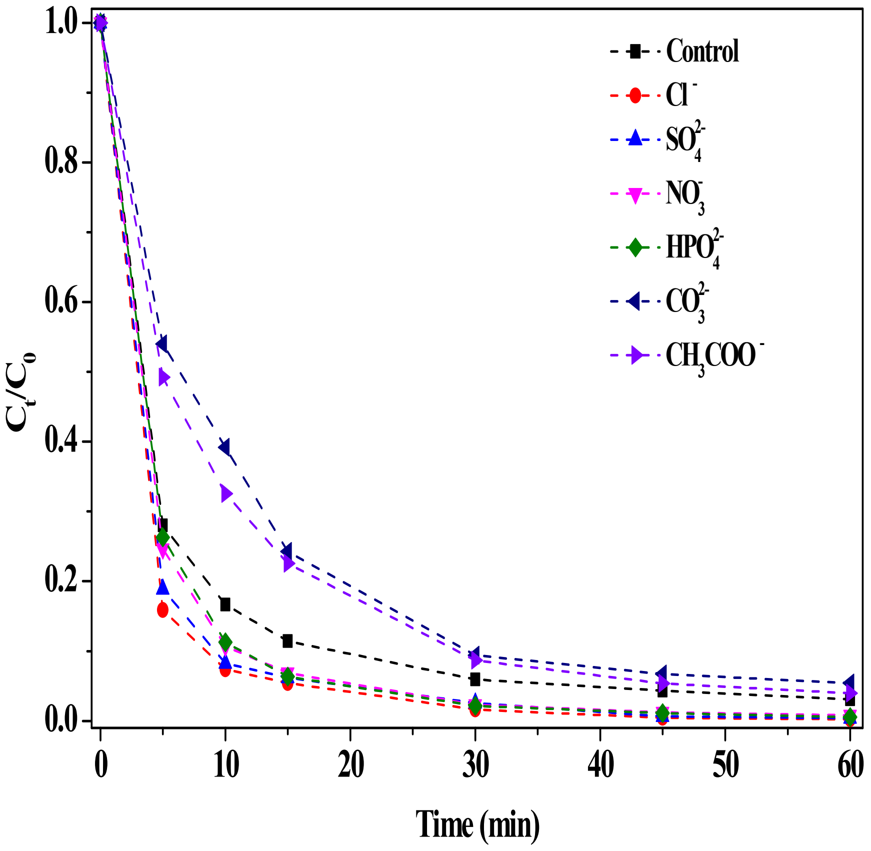

Figure S9 Effect of various anions on the RhB degradation. Reaction conditions: [$NaHCO_3$] = 10 mM, [H_2O_2] = 15 mM, [O/g-C_3N_4] = 0.8 g L^{-1}, [Anions] = 100 mM, [RhB] = 10 mg L^{-1}, 25 °C, pH ≈ 8.4 and 60 min.

Figure S10

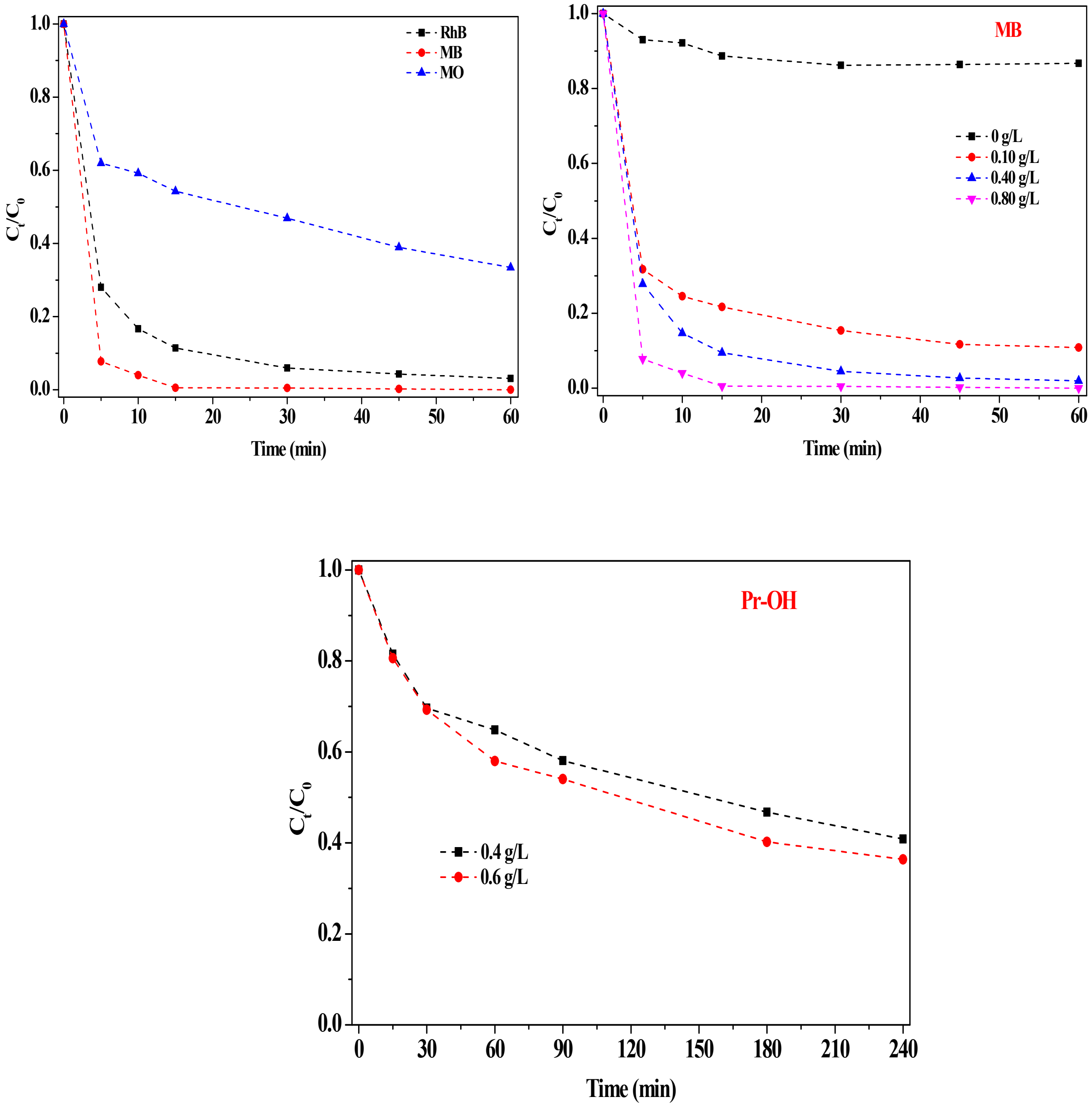

Figure S10 Effect of other organic pollutants on the degradation. Reaction conditions: $[NaHCO_3]$ = 10 mM, $[H_2O_2]$ = 15 mM, $[O/g\text{-}C_3N_4]$ = 0.8 g L^{-1}, [Organics] = 10 mg L^{-1}, 25 ^{o}C, pH ≈ 8.4 and 60 min.

Figure S11

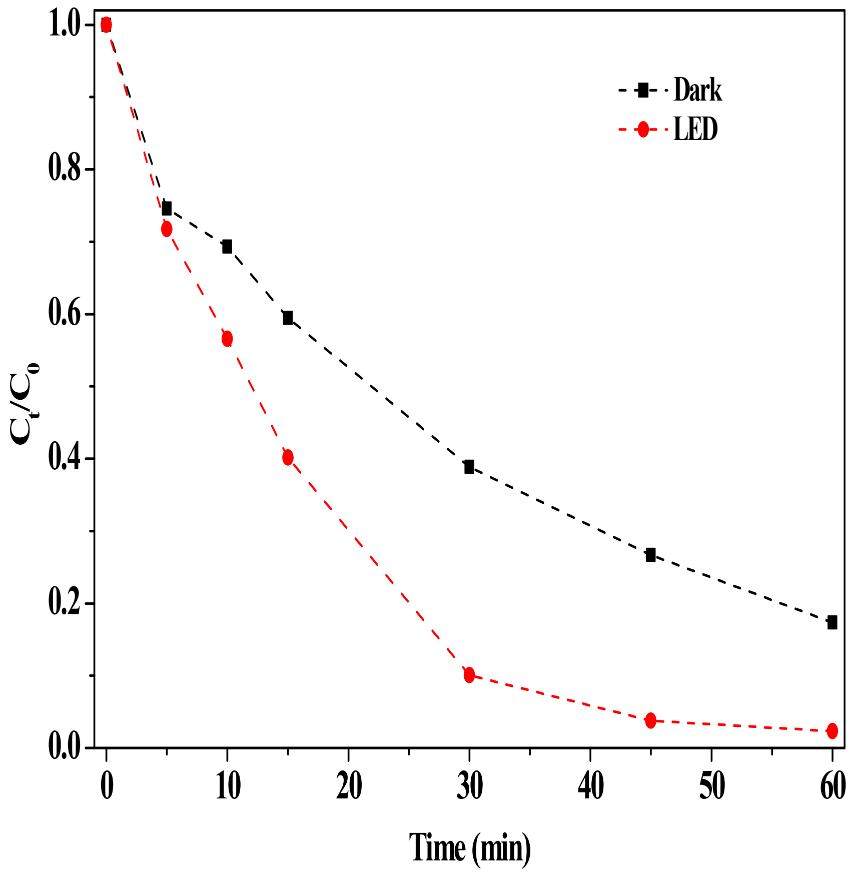

Figure S11 Comparison results of dark and LED illumination on the RhB degradation. Reaction conditions: [$NaHCO_3$] = 10 mM, [H_2O_2] = 15 mM, [O/g-C_3N_4] = 0.1 g L^{-1}, [RhB] = 10 mg L^{-1}, 25 °C, pH ≈ 8.4 and 60 min.

.

Figure S12

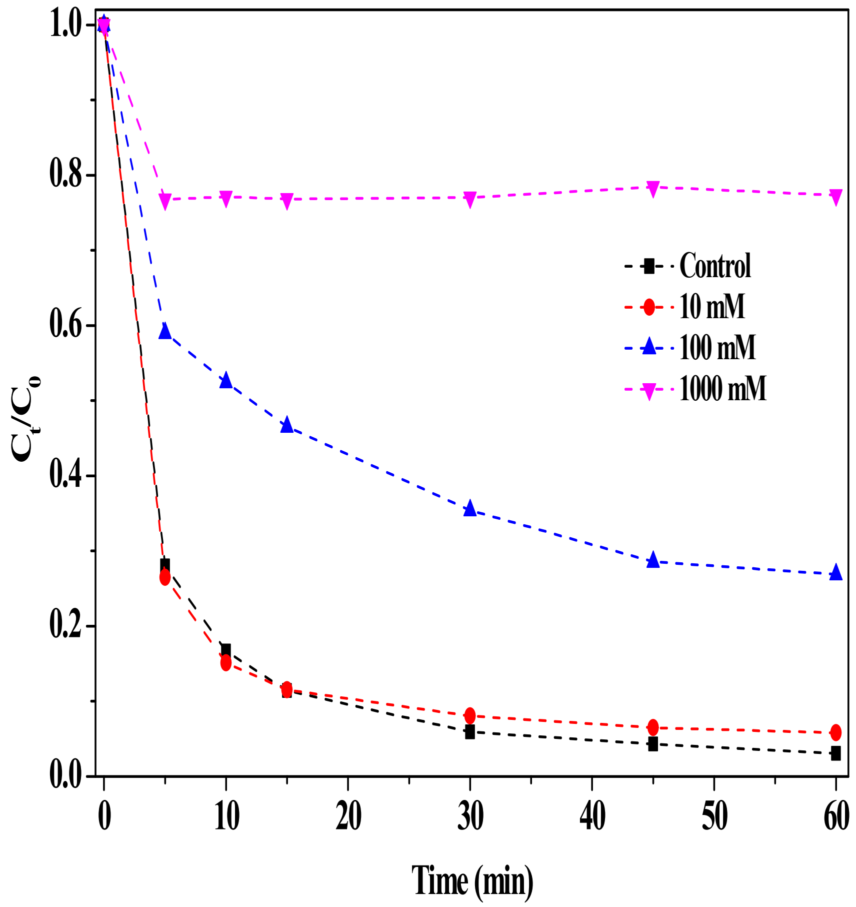

Figure S12 Effect of concentrations of ascorbic acid on the RhB degradation. Reaction conditions: [$NaHCO_3$] = 10 mM, [H_2O_2] = 15 mM, [O/g-C_3N_4] = 0.8 g L^{-1}, [RhB] = 10 mg/L, 25 ^oC, pH ≈ 8.4, 60 min, [Ascorbic acid] = 10, 100 and 1000 mM, respectively.

Figure S13

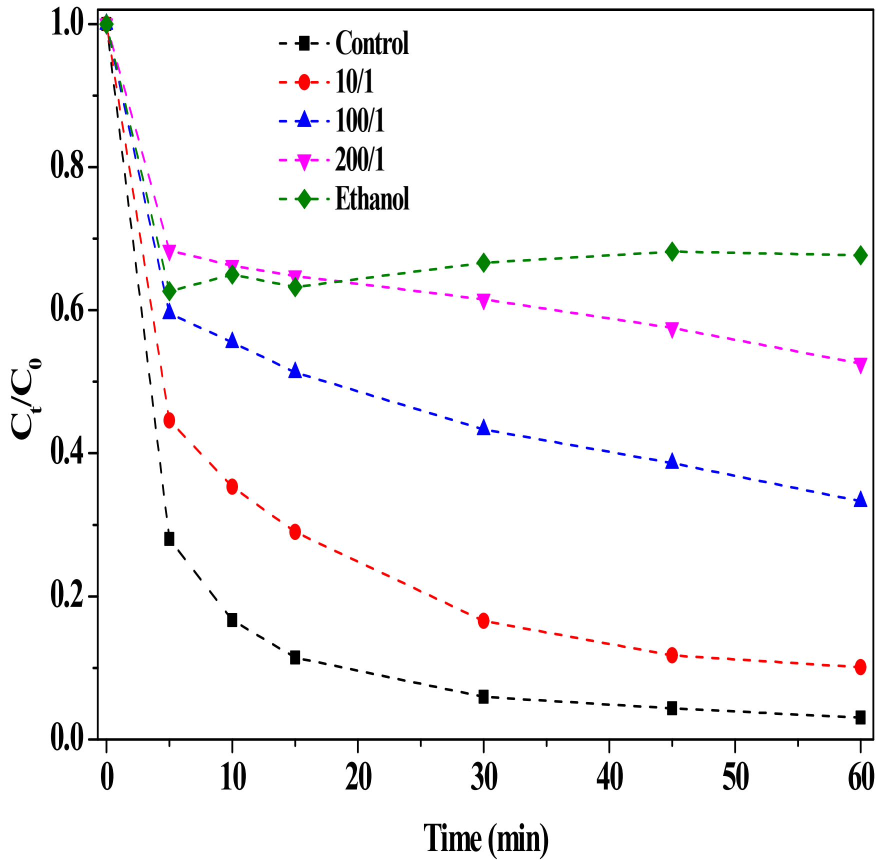

Figure S13 Effect of concentrations of ethanol on the RhB degradation. Reaction conditions: [$NaHCO_3$] = 10 mM, [H_2O_2] = 15 mM, [O/g-C_3N_4] = 0.8 g L^{-1}, [RhB] = 10 mg L^{-1}, 25 ^{o}C, pH ≈ 8.4, 60 min, [Ethanol] = 0.15 M, 1.5 M, 3.0 M, ethanol replacing of water, respectively.

Figure S14

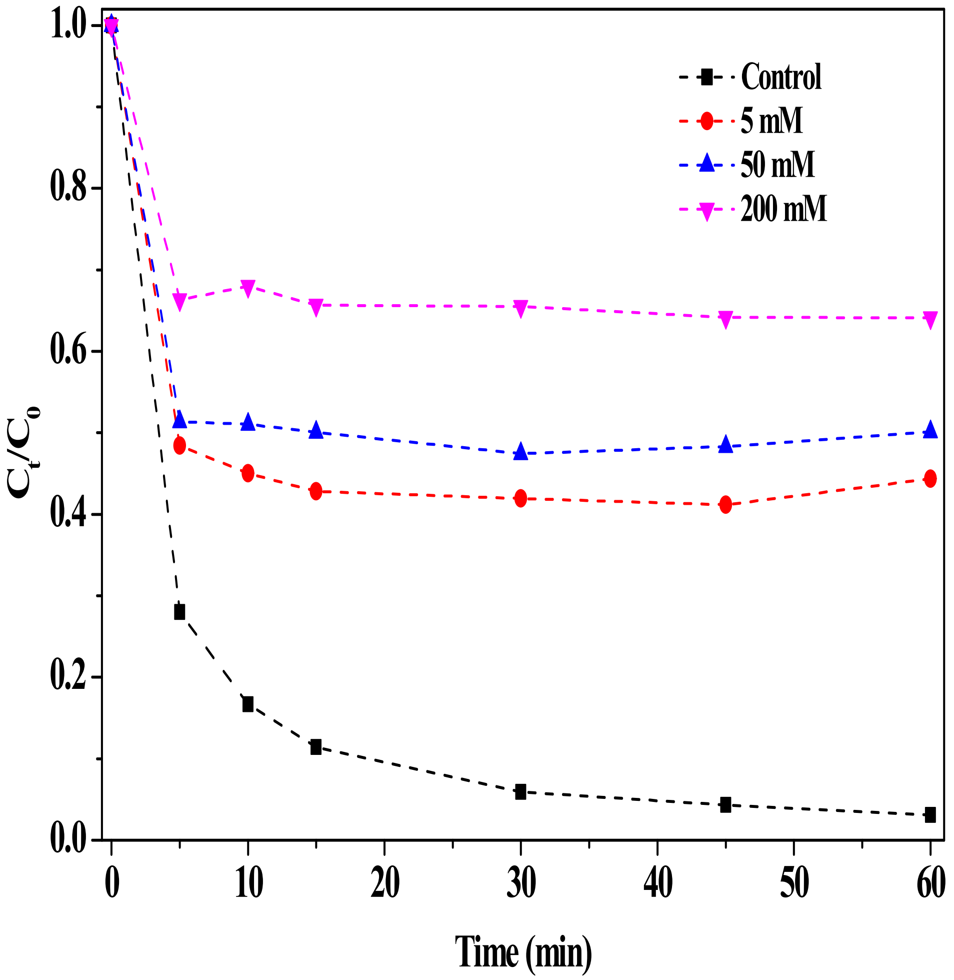

Figure S14 Effect of concentration of Tiron on the RhB degradation. Reaction conditions: $[NaHCO_3]$ = 10 mM, $[H_2O_2]$ = 15 mM, $[O/g\text{-}C_3N_4]$ = 0.8 g L^{-1}, [RhB] = 10 mg L^{-1}, 25 ^oC, pH ≈ 8.4, 60 min, [Tiron] = 5, 50 and 200 mM, respectively.

Figure S15

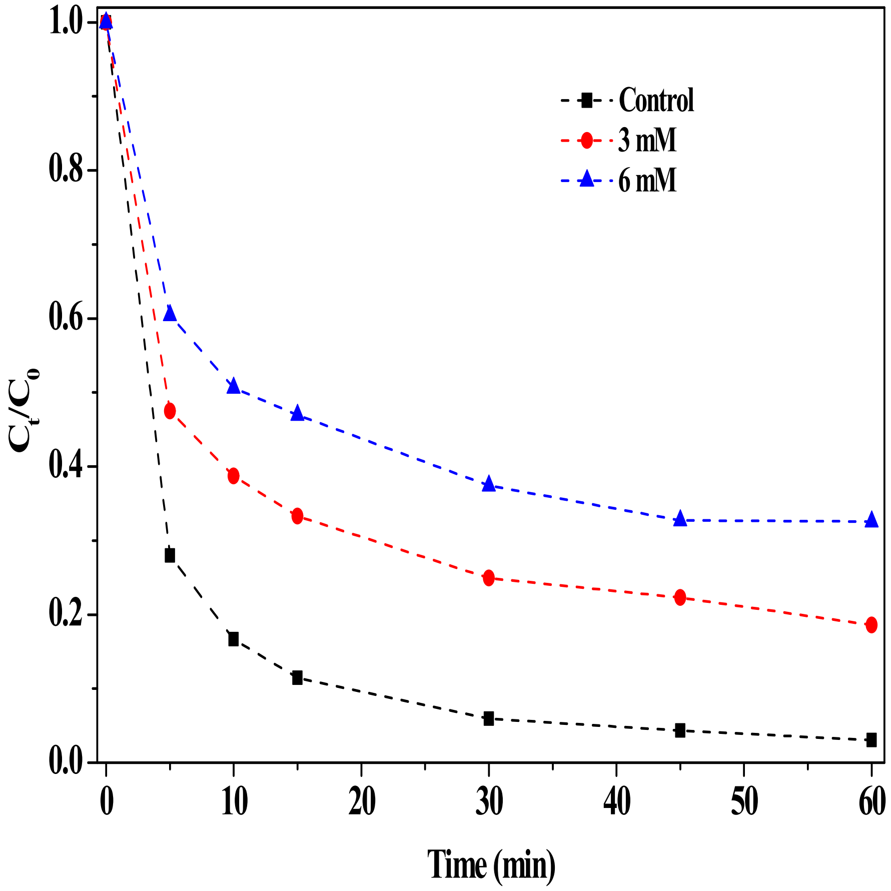

Figure S15 Effect of concentration of benzoquinone (BQ) on the RhB degradation. Reaction conditions: [$NaHCO_3$] = 10 mM, [H_2O_2] = 15 mM, [O/g-C_3N_4] = 0.8 g L^{-1}, [RhB] = 10 mg L^{-1}, 25 ^{o}C, pH ≈ 8.4, 60 min, [BQ] = 3 and 6 mM, respectively.

Figure S16

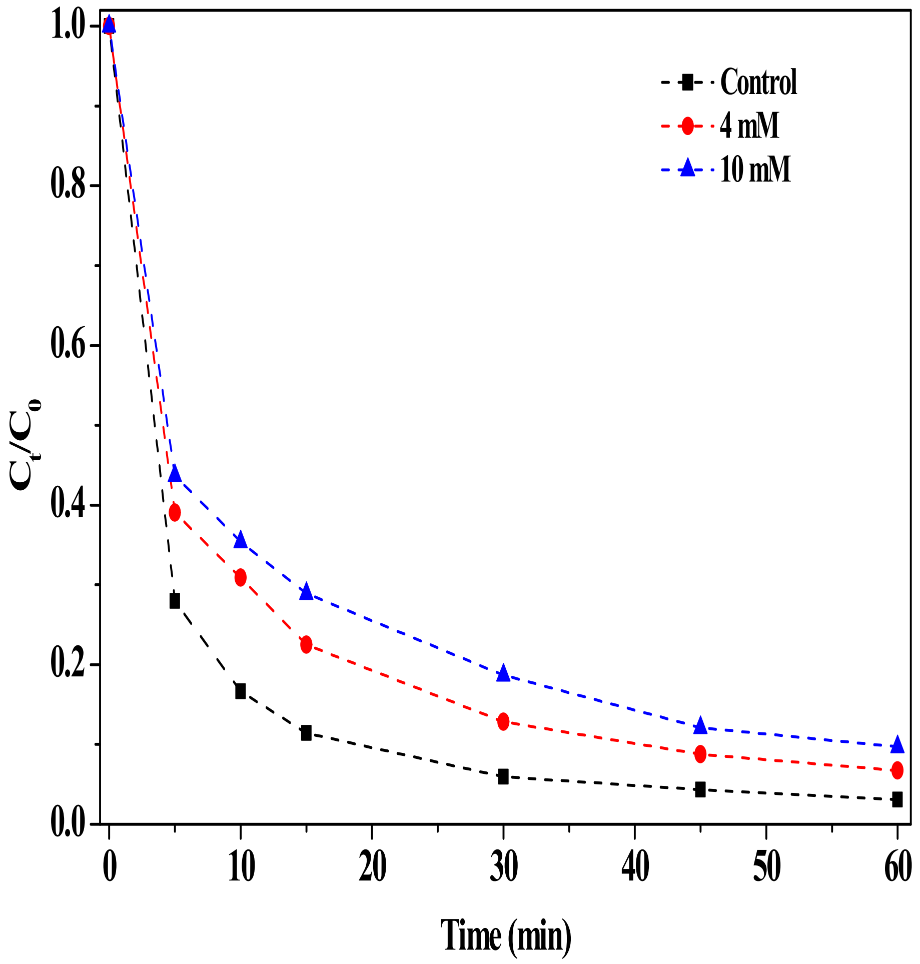

Figure S16 Effect of concentration of NaN_3 on the RhB degradation. Reaction conditions: [$NaHCO_3$] = 10 mM, [H_2O_2] = 15 mM, [O/g-C_3N_4] = 0.8 g L^{-1}, [RhB] = 10 mg L^{-1}, 25 ^{o}C, pH ≈ 8.4, 60 min, [NaN_3] = 4 and 10 mM, respectively.

Figure S17

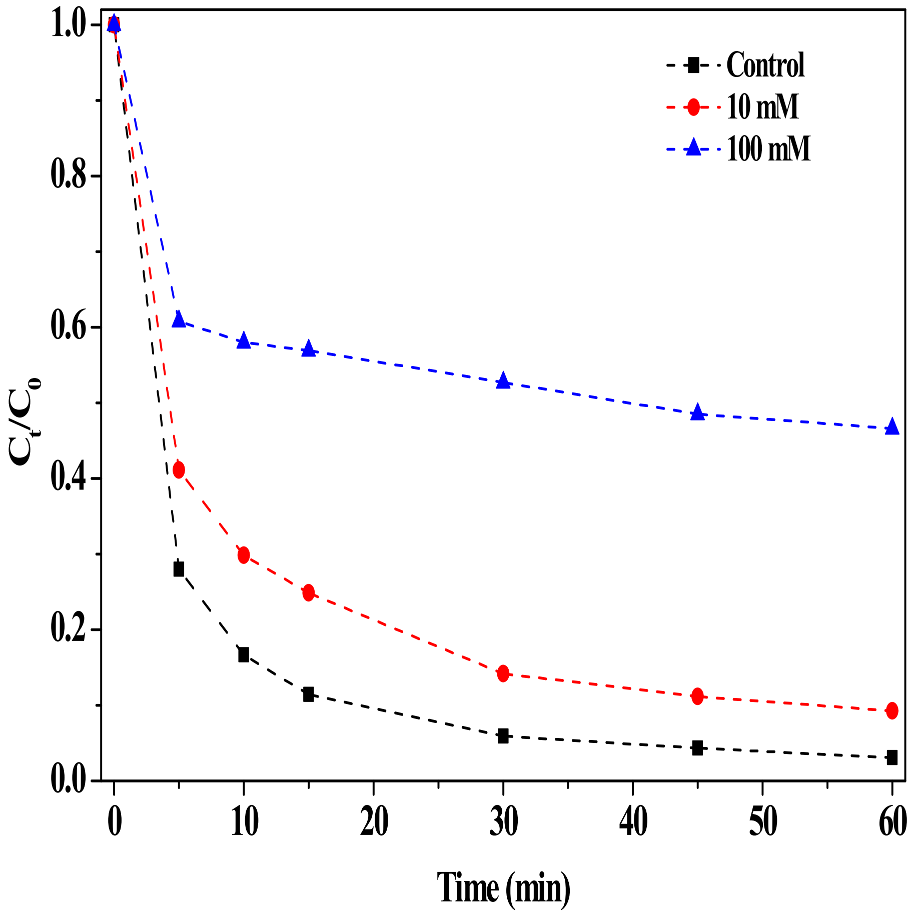

Figure S17 Effect of concentrations of furfuryl alcohol (FFA) on the RhB degradation. Reaction conditions: [$NaHCO_3$] = 10 mM, [H_2O_2] = 15 mM, [O/g-C_3N_4] = 0.8 g L^{-1}, [RhB] = 10 mg L^{-1}, 25 ^{o}C, pH ≈ 8.4, 60 min, [FFA] = 10 and 100 mM, respectively.

Figure S18

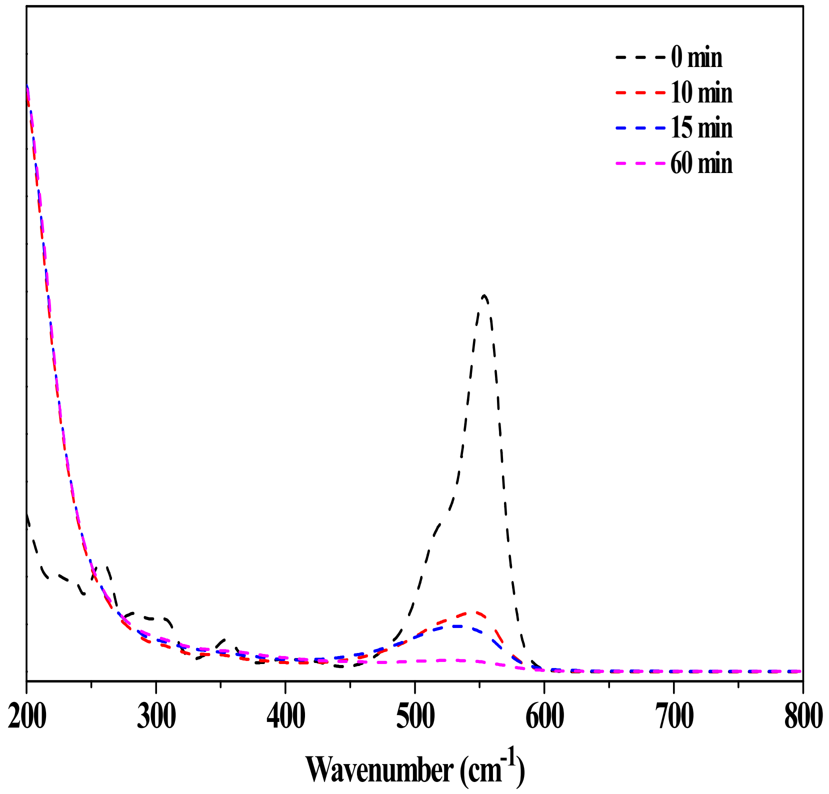

Figure S18 The degradation for RhB with the increasing time from UV-vis results.

Figure S19

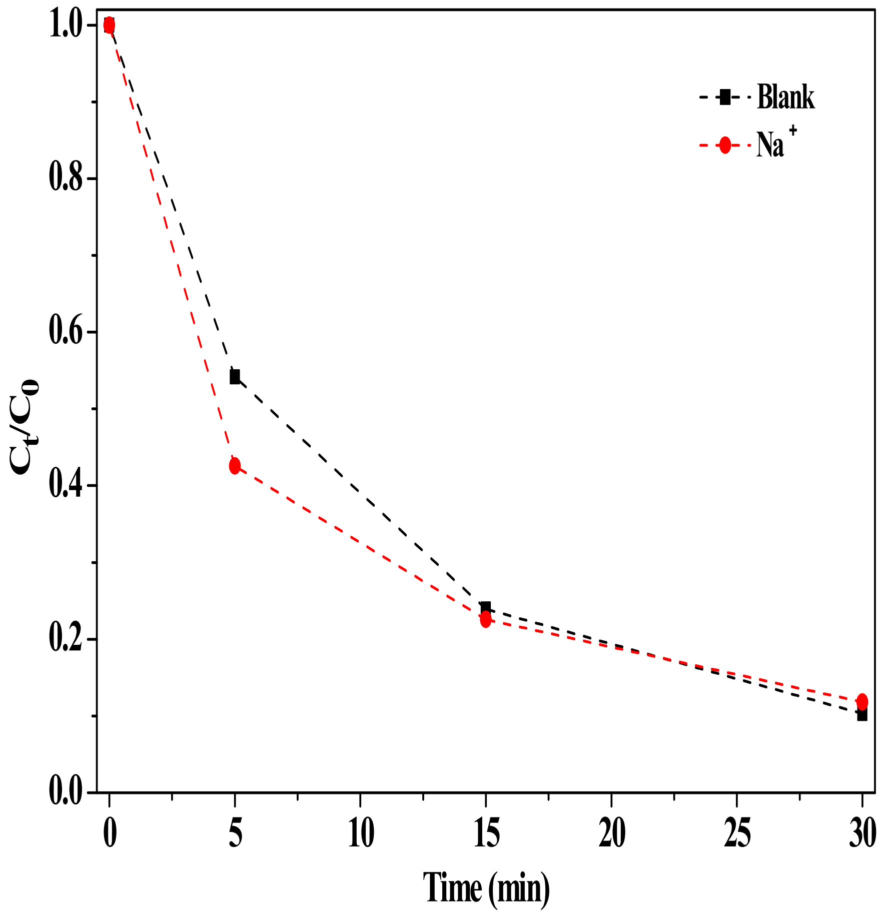

Figure S19 The effect of Na^+ over different catalysts for the RhB degradation. Reaction conditions: $[NaHCO_3]$ = 10 mM, $[H_2O_2]$ = 15 mM, [Na-O/g-C_3N_4 or O/g-C_3N_4] = 0.8 g L^{-1}, [RhB] = 10 mg L^{-1}, 25 ^oC, pH ≈ 8.4 and 30 min.

Figure S20

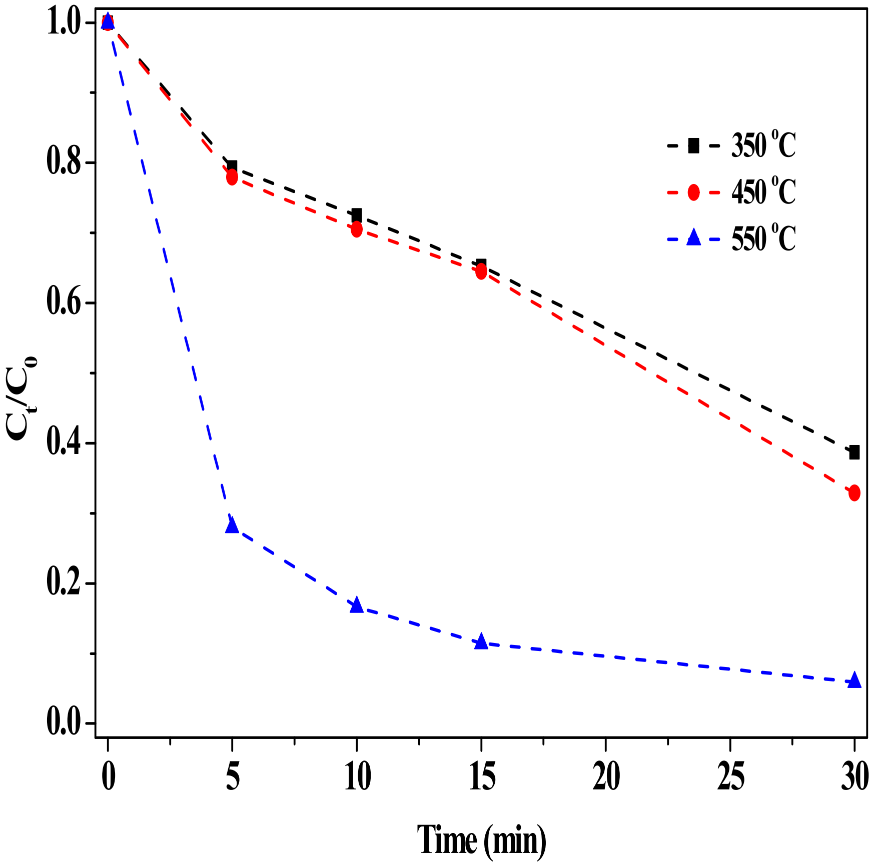

Figure S20 Effect of calcination temperature of O/g-C_3N_4 on the RhB degradation. Reaction conditions: [$NaHCO_3$] = 10 mM, [H_2O_2] = 15 mM, [O/g-C_3N_4] = 0.8 g L^{-1}, [RhB] = 10 mg L^{-1}, 25 °C, pH ≈ 8.4 and 60 min.

Table S1 Results of the content in g-C_3N_4 and O/g-C_3N_4 catalysts from element analysis.

Catalysts	C(wt%)	N(wt%)	O(wt%)	mol(C)/(N)
g-C_3N_4	36.39	62.49	1.13	0.68
O/g-C_3N_4	38.77	59.20	2.02	0.76

Table S2 The results of ratio of N(sp^2) and N(sp^3) and degradation of RhB in 30 min.

Catalysts	N(sp^2)/N(sp^3)	Degradation efficiency(%)
O/g-C_3N_4-350 ^oC	1.98	61.3
O/g-C_3N_4-450 ^oC	2.06	67.1
O/g-C_3N_4-550 ^oC	1.74	94.1
